# Structure-Based Prediction and Ranking of PFAS Groundwater Mobility for EU Drinking Water Regulation

**DOI:** 10.1021/acs.est.6c11513

**Published:** 2026-09-29

**Authors:** Sergio de-la-Huerta-Sainz, Ozge Ozkilinc, Valentín Diez-Cabanes, María Antonieta Escobedo-Monge, Pedro A. Marcos, Alfredo Bol-Arreba, Mert Atilhan, Rocio Barros, Santiago Aparicio

**Affiliations:** † Department of Chemistry, 16725 University of Burgos, Burgos 09001, Spain; ‡ ICCRAM (International Research Centre in Critical Raw Materials and Advanced Industrial Technologies), Burgos 09001, Spain; § Department of Physics, University of Burgos, Burgos 09001, Spain; ∥ Department of Chemical and Paper Engineering, 4175 Western Michigan University, Kalamazoo, Michigan 49008-5462, United States

**Keywords:** PFAS, groundwater mobility, machine learning, XGBoost, SHAP, EU Drinking Water Directive, QSPR, soil sorption, forever chemicals, risk prioritization

## Abstract

The EU Drinking Water Directive (2020/2184) mandates monitoring of 20 priority per- and polyfluoroalkyl substances (PFAS) from January 2026, yet predicting which PFAS structures pose the greatest groundwater contamination risk remains challenging. We present a machine learning framework that simultaneously predicts the three physicochemical properties governing PFAS subsurface mobility—soil sorption (log KOC, R^2^ = 0.90), water solubility (log Sw, R^2^ = 0.90), and ionization class (93% accuracy)—from molecular structure alone. The models were trained on 122 PFAS spanning 37 subclasses using 44 molecular descriptors, including nine that encode the segmentation of the fluorinated backbone by ether linkages, and were validated by cross-validation, external test sets, leave-one-subclass-out analysis, Y-randomization, and a formal applicability domain. SHAP analysis indicates that ether-linkage descriptors are associated with lower predicted soil retention, providing a model-based explanation for the high mobility of GenX-type replacements. A composite Groundwater Mobility Index integrating the three end points identifies PFBA, PFPeA, and PFPeS as the most mobile EU-regulated compounds, consistent with field monitoring evidence, and flags several nonregulated PFAS with comparable mobility. An open-access prediction tool with applicability-domain screening accompanies this work.

## Graphical abstract

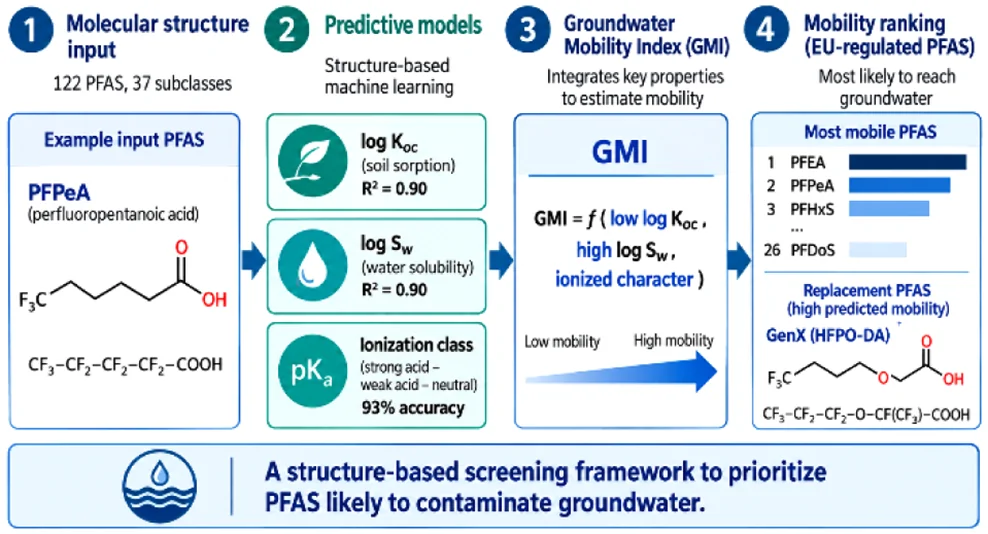

## 1 Introduction

Per- and polyfluoroalkyl substances (PFAS) constitute a family of synthetic chemicals characterized by the extraordinary strength of the carbon–fluorine bond, which renders them resistant to thermal, chemical, and biological degradation.^1,2^ This persistence, combined with widespread industrial use since the 1950s in products ranging from firefighting foams to food-contact materials, has resulted in ubiquitous environmental contamination.^3,4^ PFAS have been detected in surface waters, groundwater, soils, sediments, biota, and drinking water supplies across all continents.^5,6^ The health implications of chronic PFAS exposure—including immunotoxicity, endocrine disruption, hepatotoxicity, and associations with certain cancers—have placed these substances at the center of a global regulatory effort.^7,8^

The European Union has responded with progressively stringent measures. The recast Drinking Water Directive (DWD, EU 2020/2184) introduced two PFAS parameters enforceable from January 2026: a “PFAS Total” limit of 0.50 μg/L and a “Sum of PFAS” limit of 0.10 μg/L, the latter defined as the sum of 20 individual perfluoroalkyl acids (10 perfluorocarboxylic acids and 10 perfluorosulfonic acids) spanning chain lengths C4–C13.^9,10^ Complementary restrictions under REACH target PFOA, PFOS, PFHxS, PFHxA, and C9–C14 PFCAs, while a proposal for a universal PFAS restriction covering approximately 10,000 substances is under evaluation by ECHA.^11,12^ The regulatory ambition is clear: reduce PFAS emissions to protect drinking water quality. What is less clear is which of the thousands of PFAS in commerce or the environment are most likely to contaminate groundwater—that is, which molecular structures translate into high environmental mobility.

The composition of the Annex III list reflects a specific regulatory history rather than an assessment of subsurface mobility, and this distinction is central to the present work. The list comprises the C4–C13 homologues of perfluoroalkyl carboxylic and sulfonic acids. That scope was determined by two considerations: the analytical methods that had been standardized and interlaboratory validated at the time of drafting, and the 2020 EFSA CONTAM opinion, which derived a group tolerable weekly intake from immunotoxicity data available for four substances (PFOA, PFNA, PFHxS and PFOS) with sufficient human epidemiological coverage.8,9 Neither criterion—analytical tractability and toxicological data availability—measures the propensity of a compound to migrate through soil and reach an abstraction well. Substances outside the perfluoroalkyl acid domain are therefore absent from the list irrespective of their subsurface behavior, including the ether-based replacements HFPO–DA (GenX) and ADONA and the ultrashort-chain acid trifluoroacetic acid, all three of which are documented groundwater contaminants.2,6 The gap between what is regulated and what is mobile is the specific problem this work addresses, and it is the reason a structure-based ranking is required rather than a list-based one.

The environmental fate of PFAS in the subsurface is governed principally by three physicochemical properties.^13,14^ First, the soil organic carbon–water partition coefficient (K_OC_) determines the degree to which a compound is retained by soil organic matter; low K_OC_ implies weak retention and rapid leaching.^15,16^ Second, water solubility (S_w_) controls the maximum dissolved-phase concentration available for transport; highly soluble PFAS remain in the aqueous phase and migrate with infiltrating water.^17^ Third, the acid dissociation constant (p*K*_a_) governs speciation at environmental pH, which in turn affects both solubility and sorption: fully ionized (anionic) PFAS are generally more mobile than neutral species.^18,19^ Together, these three parameters define the intrinsic groundwater mobility of a PFAS molecule.

Experimental determination of these properties is laborious and, for many PFAS, simply unavailable. The ITRC’s physical–chemical property compilation covers approximately 80 substances, and reliable experimental K_OC_ values exist for fewer than 50.^20^ Predictive models offer a way forward. Quantitative structure–property relationship (QSPR) approaches have been applied to PFAS, notably using COSMO-RS for partition coefficients^21,22^ and OPERA for a range of fate end points.^23^ However, these tools were largely developed on nonfluorinated training sets and often perform poorly for PFAS, particularly for ionizable compounds.^24,25^ Machine learning (ML) has shown promise in overcoming these limitations. Recent studies have applied ML to predict PFAS soil sorption (K_d_),^26–28^ aquatic toxicity,^29^ degradation rates,^30^ and bioaccumulation.^31^ A notable contribution is the PFASorptionML tool, which predicts K_d_ from PFAS and soil properties using a data set of 1,274 entries.^26^ Yet three fundamental limitations persist across this body of work. First, existing models predict single end points in isolation—sorption or solubility or toxicity—whereas groundwater mobility is a multiproperty phenomenon that cannot be captured by any one parameter alone. Second, all current tools require either soil-specific input data (limiting their use to characterized sites) or were trained on predominantly nonfluorinated compounds (limiting their applicability to PFAS). Third, no study has translated predicted physicochemical properties into a regulatory-relevant screening metric that directly informs compliance prioritization under the EU Drinking Water Directive. The result is that regulators and water authorities lack a practical, structure-based tool to answer the question that matters most: given a PFAS molecular structure, how likely is it to reach drinking water?

Here we address this knowledge gap. We compiled a curated database of 122 PFAS covering 37 structural subclasses—from legacy perfluoroalkyl acids to replacement ether compounds such as HFPO–DA (GenX), fluorotelomer derivatives, sulfonamides, zwitterionic and cationic PFAS, and branched isomers—with documented values for log K_OC_, p*K*_a_, and log S_w_. We developed a multioutput XGBoost framework that simultaneously predicts soil sorption (regression), water solubility (regression), and ionization class (3-class classification) from a final set of 44 molecular descriptors. The models were validated by 5-fold cross-validation, external test sets, and leave-one-subclass-out (LOSO) analysis. SHAP analysis was used to identify the molecular features controlling each end point. Finally, we combined the three model outputs into a Groundwater Mobility Index (GMI) that ranks all 122 PFAS—including the 20 EU DWD priority substances—by their intrinsic potential to reach drinking water. An open-access prediction tool accompanies this work. Together, these elements constitute the first framework that connects molecular structure directly to a regulatory-relevant groundwater mobility ranking—requiring no experimental input, no soil characterization, and no computational chemistry beyond descriptor calculation from a SMILES string.

The novelty of this work lies in three specific advances rather than in the application of machine learning to PFAS as such, which is now well established.26–31 First, existing models predict a single end point: PFASorptionML returns *K*_d_ but requires measured soil properties as input;26 OPERA and EPI Suite return solubility or partitioning but were parametrized predominantly on nonfluorinated chemicals;23 COSMOtherm returns accurate partition coefficients but requires quantum-chemical conformer ensembles per compound.17 None returns the joint property profile that determines mobility. Second, no published tool converts predicted properties into a ranked, decision-ready metric; the integration step is presently left to the user. Third, and consequently, no structure-only screening resource exists that a water authority can apply to a newly detected PFAS without laboratory measurement, site characterization or computational chemistry infrastructure. Table S8 (Supporting Information) benchmarks the present framework against these tools on input requirements, end point coverage, PFAS-specific training and regulatory output.

## 2 Methods

### 2.1 Database Compilation

A systematic literature search was conducted in Web of Science, Scopus, and the EPA CompTox Chemistry Dashboard to compile physicochemical end point data for PFAS. Three target properties were collected for each compound: (i) the soil organic carbon–water partition coefficient (log K_OC_), preferring values derived from batch sorption experiments at the lowest reported PFAS concentration and at native soil pH;^15,16,26^ (ii) the acid dissociation constant (p*K*_a_), with priority given to recent NMR-based experimental values;^32^ and (iii) water solubility (log S_w_ in mg/L), from direct experimental measurement where available.^20^ When experimental data were unavailable, values were taken from COSMOtherm predictions^21^ or OPERA QSAR models via the CompTox Dashboard.^23^ Each data point is annotated with its source and quality level (experimental, COSMO-RS predicted, OPERA predicted, or estimated from homologous series trends). The database includes all 20 PFAS listed in Annex III of the EU DWD, plus 102 additional PFAS selected to maximize structural diversity. CAS numbers and SMILES notations were verified against the CompTox Dashboard. The complete database is provided as Supporting Information.

### 2.2 Molecular Descriptors

Molecular structures were encoded as SMILES strings and processed with the RDKit library (version 2024.03).^33^ A set of 35 descriptors was computed for each compound, comprising: (a) constitutional descriptors (molecular weight, atom counts for C, F, O, N, S, Cl, P, number of heavy atoms, number of H-bond donors and acceptors, number of rotatable bonds, ring count); (b) topological descriptors (Bertz complexity, Chi0v and Chi1v connectivity indices, Hall–Kier alpha, Kappa shape indices); (c) electronic descriptors (topological polar surface area, maximum and minimum partial charges, fraction CSP3); (d) lipophilicity descriptors (Wildman–Crippen MolLogP, molar refractivity); (e) molecular size (Labute accessible surface area); and (f) PFAS-specific binary and ratio descriptors (fluorine-to-heavy-atom ratio, F/C ratio, estimated CF_2_ units, and binary indicators for sulfonate, carboxylate, ether, nitrogen, phosphorus, and chlorine functionality). No target end point values were included as features, ensuring a strictly structure-based prediction. Initial LOSO testing (Section 3.3) revealed that this descriptor set failed to generalize to perfluoroalkyl ether carboxylic acids, indicating that it did not encode what makes ether-linked PFAS chemically distinct. The set was therefore extended with nine descriptors that explicitly represent the segmentation of the fluorinated backbone by ether oxygens: the length of the longest contiguous perfluoroalkyl segment, the number and mean length of such segments and their standard deviation, the ether-oxygen-to-carbon and total-oxygen-to-carbon ratios, the number of terminal CF3 groups, the topological bond distance from the acid headgroup to the nearest ether oxygen, and the count of branch points adjacent to an ether oxygen. These quantities are computed directly from the molecular graph by cutting the carbon skeleton at ether linkages and measuring the resulting fluorinated fragments. They are motivated by the physical argument that an ether oxygen interrupts the contiguous hydrophobic surface that drives partitioning to organic matter, so that a C8 ether acid does not behave as a C8 perfluoroalkyl acid but as two shorter segments. The final descriptor set therefore comprises 44 variables. All descriptors are listed with definitions in Table S3 (Supporting Information).

No fixed compound-to-descriptor ratio is prescribed for regularized gradient-boosted tree ensembles, in contrast to ordinary least-squares regression where the classical 5:1 guideline originates and where the parameter count grows directly with the descriptor count. Tree ensembles do not fit one coefficient per descriptor; the effective model complexity is governed by tree depth, the number of trees, and the L1/L2 penalties, and descriptors that carry no signal are simply never selected at a split. We nevertheless tested the concern directly in two ways. A descriptor-count ablation (Figure S8, Supporting Information) shows that predictive performance rises steeply to approximately 15 descriptors and then plateaus: for log KOC, a 15-descriptor subset achieves R^2^ = 0.897 against R^2^ = 0.911 for the full set, so the model is not dependent on the larger descriptor count, and the additional variables refine rather than create the fit. A Y-randomization test (Figure S7, Supporting Information) provides the formal chance-correlation control: with the descriptor matrix held fixed and the response vector permuted 100 times, the mean cross-validated R^2^ falls to −0.52 for log KOC and −0.47 for log Sw, with maximum permuted values of −0.13 and −0.15 respectively. No permuted model approached the true performance (Z = 9.5 and 9.2; empirical *p* < 0.01). Chance correlation can therefore be excluded as an explanation for the reported accuracy.

### 2.3 p*K*_a_ Classification

Rather than treating p*K*_a_ as a continuous regression target—which is problematic for PFAS given the bimodal distribution between strong acids (p*K*_a_ < 1 for PFCAs and PFSAs) and neutral compounds (p*K*_a_ ≥ 7 for FTOHs and FASEs)—we adopted a three-class classification scheme grounded in environmental chemistry: (i) **strong acid** (p*K*_a_ < 1): essentially fully ionized under typical environmental pH conditions and present predominantly as anions; (ii) **weak acid** (1 ≤ p*K*_a_ < 7): partially ionized, with pH-dependent speciation affecting mobility; (iii) **neutral/high-pKa class** (p*K*_a_ ≥ 7): treated operationally as the weakly/non-ionized class in the model; ionization may increase as environmental pH approaches or exceeds the compound pKa. This classification directly informs environmental behavior without requiring precise p*K*_a_ values, which remain uncertain for many PFAS.^32,34^

### 2.4 Machine Learning Models

Three XGBoost models were trained independently: two regressors for log K_OC_ and log S_w_, and one multiclass classifier for the p*K*_a_ class. The data set was split into training (80%) and test (20%) sets using stratified sampling on the p*K*_a_ class to ensure balanced representation. Hyperparameters were set as follows: 400 trees, maximum depth 4, learning rate 0.06, subsample fraction 0.85, column subsample 0.75, L1 regularization (α) 0.15, and L2 regularization (λ) 1.5. The classifier used the XGBoost multi:softprob objective for multiclass probability estimation These values were selected through preliminary grid search with 5-fold cross-validation to balance predictive accuracy against overfitting risk. All models were implemented using XGBoost version 3.2.0 and interpreted with SHAP version 0.51.0, with scikit-learn 1.8.0 for validation protocols, under Python 3.12.

### 2.5 Model Validation

Three validation protocols were applied. (i) 5-fold cross-validation (CV) on the training set, reporting R^2^, MAE, and RMSE for regression models, and accuracy, balanced accuracy, and weighted F1 score for the classifier. (ii) External test set evaluation on the held-out 20% of compounds. (iii) Leave-one-subclass-out (LOSO) analysis, in which all compounds from one structural class (e.g., all PFCAs, all PFSAs, all PFECAs) were sequentially excluded from training and predicted as an external set. LOSO provides the most stringent assessment of generalizability to structurally novel PFAS. These three protocols are complementary rather than redundant, and each answers a different question that a regulatory user would ask. Random 5-fold CV estimates the error expected for a new compound drawn from the same structural population as the training data—the situation when an authority screens a further homologue of a familiar family. The held-out external set provides a single unbiased estimate on data untouched during model selection, guarding against the optimistic bias that accumulates when cross-validation is used repeatedly to guide hyperparameter choices. Leave-one-subclass-out removes an entire structural family from training and therefore simulates the harder and more policy-relevant case of a genuinely novel replacement chemistry entering commerce. This third protocol is essential specifically for PFAS because their chemical space is organized into discrete, chemically coherent subclasses rather than being distributed continuously: a random fold almost always leaves close structural analogues of every test compound in the training set, so random CV systematically overestimates performance for novel chemistries. Reporting random CV alone would misrepresent the applicability of the model to precisely the use case for which it is intended.

### 2.6 Applicability Domain

Because the framework is distributed as an open-access tool intended for compounds outside the training set, a formal applicability domain (AD) was defined so that predictions for structurally unfamiliar inputs are flagged rather than reported silently. Two complementary criteria are used. The first is the leverage h_i = x_i^T^(X^T^X)^−1^x_i evaluated on the 15 highest-ranked descriptors, with the conventional warning threshold h* = 3­(p+1)/n = 0.393; the reduced basis is required because with all 44 descriptors h* exceeds unity and the leverage criterion becomes undefined. The second is the mean Euclidean distance to the five nearest training neighbors in standardized descriptor space, with the 95th percentile of the training distribution (3.21) as threshold. A query compound is reported as inside the domain only if it satisfies both criteria; otherwise the prediction is returned with an explicit out-of-domain warning and the GMI is suppressed. The same logic is implemented in the distributed prediction tool.

### 2.7 SHAP Interpretability Analysis

SHapley Additive exPlanations (SHAP)^35^ were computed using TreeExplainer for all three models. For each compound in the training set, SHAP values quantify the contribution of each molecular descriptor to the model’s prediction, enabling mechanistic interpretation of structure–fate relationships. We report: (a) global feature importance as mean absolute SHAP values; (b) beeswarm plots showing the distribution of SHAP values colored by feature magnitude; (c) dependence plots for key descriptor–end point pairs; and (d) per-class SHAP analysis for the p*K*_a_ classifier. Pearson correlations between SHAP values and feature values were calculated to identify the directionality of structure–fate relationships.

### 2.8 Groundwater Mobility Index

To integrate the three end points into a single, interpretable metric, we defined a composite Groundwater Mobility Index (GMI) as

1 $$GMI=0.40\times norm\left(-\log K_{OC}\right)+0.35\times norm\left(\log S_{w}\right)+0.25\times I_{ion}$$

where norm­() denotes min–max normalization to [0,1], and I_ion_ is the ionization score: 1.0 for strong acids, 0.5 for weak acids, and 0.0 for neutrals, weighted by the classifier’s confidence (maximum class probability). The weights (0.40, 0.35, 0.25) were assigned by expert judgment to reflect the conventional ordering of the importance of sorption, dissolved-phase availability, and speciation in subsurface PFAS transport.^13,14^ Higher GMI values indicate greater intrinsic mobility toward groundwater. The GMI thus transforms three independent model outputs—each of which has value individually—into a single decision-relevant metric aligned with the regulatory question posed by the DWD: which PFAS should be prioritized for monitoring? Existing approaches address sorption, solubility, and ionization separately, requiring the end user to integrate disparate predictions. The GMI performs this integration explicitly, with transparent weights and full sensitivity analysis (Supporting Information, Section S1), providing a directly comparable ranking across structurally diverse PFAS. Two normalization schemes are defined. In the data set-relative scheme, norm­() denotes min–max scaling over the 122 compounds of the present database. This is transparent but has the drawback that a GMI value is meaningful only relative to this particular compound set: adding a new compound with a more extreme property value rescales the scores of all existing compounds. We quantified this effect by appending a single hypothetical ultramobile compound to the set, which shifted the normalized sorption score of existing compounds by up to 0.177 units. For a tool intended for open-ended screening this behavior is undesirable. We therefore additionally define an absolute scheme in which the end points are scaled against fixed, physically anchored bounds that span the reported envelope of PFAS behavior—log KOC ∈ [0, 7] log L kg^–1^ and log Sw ∈ [−4, 6] log mg L^–1^—with values outside these bounds clipped. Scores on the absolute scale are independent of database composition and are directly comparable between studies and over time. The two schemes give almost identical orderings (Spearman ρ = 0.999 across all 122 compounds and ρ = 1.000 within the EU-20, with an identical top-five ranking; Figure S10a, Supporting Information), so the conclusions reported below are unaffected by the choice. The absolute scheme is adopted as the primary definition throughout, and is the scheme implemented in the prediction tool.

The weights require explicit justification. We state plainly that the values 0.40, 0.35, and 0.25 are an expert-judgment allocation reflecting the conventional ordering of importance of sorption, dissolved-phase availability and speciation in subsurface PFAS transport;13,14 the cited literature supports that ordering but does not provide a quantitative derivation of these particular numbers, and we do not claim that it does. Two independent derivations were therefore performed to test whether the allocation is defensible. The first is a transport-sensitivity derivation based on the retardation factor R = 1 + ρ_*b*_ *f*_*oc*_ *K*_*OC*_/θ, in which the elasticity of solute velocity with respect to each property is evaluated over f_oc = 0.001–0.05 and normalized; this yields 0.33/0.41/0.27. The second is a Shannon-entropy weighting, a standard objective multicriteria procedure that assigns weight in proportion to the information content of each normalized component, yielding 0.19/0.15/0.66. Rankings computed with these alternative allocations correlate with the adopted weights at ρ = 0.999 and ρ = 0.914 respectively over all 122 compounds, and at ρ = 1.000 within the EU-20 in both cases (Figure S10b, Supporting Information). The physically motivated derivation therefore lands close to the adopted values, while even a markedly different objective allocation leaves the regulatory ranking unchanged. Together with the weight-perturbation analysis of Section S1, this constitutes the justification for the weights: they are expert judgment, corroborated by an independent transport-physics derivation, and demonstrated to be noncritical to the conclusions.

## 3 Results and Discussion

### 3.1 Database Characteristics

The compiled database comprises 122 PFAS spanning molecular weights from 78 to 990 g/mol, fluorine atom counts from 1 to 35, and chain lengths from C1 to C20 (Supporting Information). The 37 structural subclasses include perfluoroalkyl carboxylic acids (PFCAs, n = 16), perfluoroalkyl sulfonic acids (PFSAs, n = 13), perfluoroalkyl ether carboxylic acids (PFECAs, n = 15), fluorotelomer alcohols (FTOHs, n = 5), fluorotelomer sulfonates (FTS, n = 4), sulfonamides (FOSAs, n = 6), sulfonamidoacetic acids (FOSAAs, n = 4), phosphonic and phosphinic acids (PFPAs/PFPiAs, n = 6), zwitterionic and cationic PFAS (n = 7), branched isomers (n = 5), and various other subclasses. The three target end points span 8.1 log units for log K_OC_ (0.10–8.20), 11.1 log units for log S_w_ (−4.80 to 6.30 log mg/L), and three discrete classes for p*K*_a_ (61 strong acids, 31 weak acids, 30 neutral compounds). Of the 122 entries, 17 have experimentally measured log K_OC_ values, 17 have NMR or experimental p*K*_a_, and 15 have experimentally determined solubilities. The near-equal distribution across p*K*_a_ classes (61/31/30) is favorable for classification training, reducing the risk of bias toward the majority class. The wide dynamic range of both regression end points—exceeding 8 log units for KOC and 11 for Sw—provides a stringent test of model generalization: a model that performs well across such a range is unlikely to be overfitting to a narrow region of chemical space.

### 3.2 Model Performance

Figure 1 summarizes model performance. The log KOC regressor achieved R^2^ = 0.90 (MAE = 0.35 log units) in 5-fold CV and R^2^ = 0.94 (MAE = 0.29) on the held-out test set (n = 25). These errors are well within the typical experimental variability of batch sorption measurements, which commonly exceeds 0.3 log units.15,16 The log Sw model performed comparably, with CV R^2^ = 0.90 (MAE = 0.54 log units) and test R^2^ = 0.90 (MAE = 0.53). For context, the OPERA water solubility model reports R^2^ = 0.87 on its internal validation, but its training set was dominated by nonfluorinated compounds.23 The p*K*_a_ classifier achieved 93% accuracy (balanced accuracy 90%, weighted F1 = 0.93) in CV and 92% on the test set, with perfect classification of strong acids and strong performance for weak acids and neutrals (Figure 1c). Because the test set contains only 25 compounds, the test-set metrics carry appreciable sampling uncertainty and should be read alongside the cross-validated values rather than in place of them. There is no systematic gap between CV and test metrics for any model, indicating the absence of overfitting; this is corroborated by the Y-randomization and descriptor-ablation analyses reported in Section 2.2.

**Figure 1:**
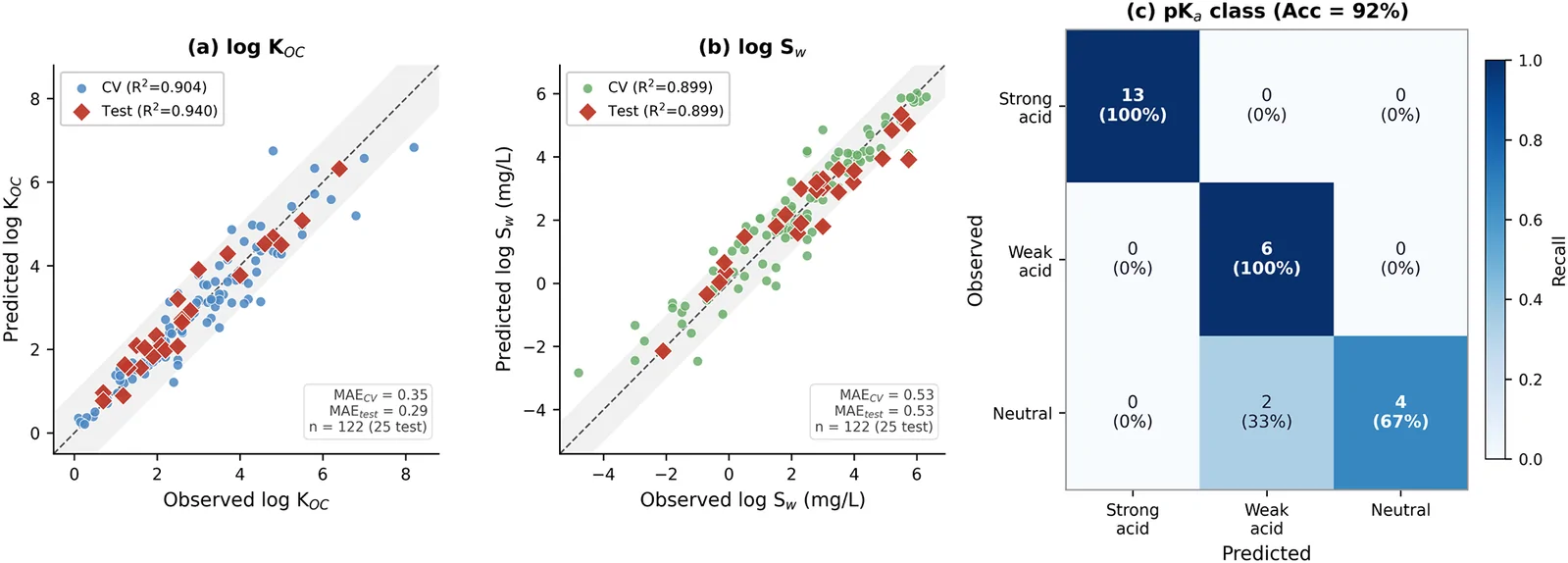
Model performance summary for the final 44-descriptor model. (a) Parity plot for log KOC predictions showing 5-fold cross-validation (blue circles) and the external test set (red diamonds); the shaded band marks ±1 log unit. (b) Parity plot for log Sw predictions showing 5-fold cross-validation (green circles) and the external test set (red diamonds). (c) Confusion matrix for the p*K*_a_ three-class classifier (strong acid/weak acid/neutral) on the external test set, with absolute counts and row-normalized percentages; color encodes recall. The test set comprises 25 compounds, so test-set metrics carry appreciable sampling uncertainty and should be read alongside the cross-validated values.

Comparison with existing tools is relevant. The PFASorptionML model^26^ predicts K_d_ (not K_OC_) and requires soil properties as input. A recent LightGBM model for PFAS sorption achieved external test R^2^ = 0.72.^28^ Our approach differs fundamentally: it predicts intrinsic compound properties from molecular structure alone, without requiring soil data, making it applicable to any PFAS regardless of the receiving environment. This distinction positions the model as a screening tool for risk prioritization rather than a site-specific sorption predictor.

### 3.3 Leave-One-Subclass-Out Validation

LOSO analysis (Table 1) provides the most rigorous test of the model’s ability to generalize to structurally novel PFAS. When all PFCAs were excluded from training, the model predicted their log KOC with R^2^ = 0.84, confirming that the chain-length–sorption relationship learned from other subclasses transfers well. PFSAs (R^2^ = 0.86), FTOHs (R^2^ = 0.91), FOSAs (R^2^ = 0.90) and FTS (R^2^ = 0.72) were similarly well predicted, giving a mean held-out R^2^ of 0.76 across the six subclasses. The principal weakness remains the PFECAs (R^2^ = 0.30), which are discussed in detail below. This behavior is consistent with the known anomalous partitioning of ether-PFAS, which do not follow the conventional KOC–KOW correlations.22,25 For the p*K*_a_ classifier, LOSO accuracy averaged 84% across subclasses, with perfect prediction for PFSAs, PFECAs and FTOHs. The lowest accuracy was obtained for FOSAs (50%), reflecting the loss of the only sulfonamide reference for the neutral class when that subclass is withheld. The perfluoroalkyl ether carboxylic acids require separate discussion because they are simultaneously the weakest subclass under LOSO and among the most policy-relevant, being the principal replacements for the long-chain acids they succeeded. With the original 35-descriptor set the held-out PFECA coefficient of determination was close to zero, indicating that the descriptors did not encode the structural feature that distinguishes these compounds. Adding the nine chain-topology descriptors described in Section 2.2 raised the held-out PFECA R^2^ from 0.10 to 0.30 and reduced the mean absolute error from 0.43 to 0.35 log units, while the mean LOSO R^2^ across the six subclasses of Table 1 rose from 0.68 to 0.76 for log KOC and from 0.37 to 0.47 for log Sw, with no loss of global cross-validated accuracy (Figure S6a,b, Supporting Information; Table 1). The improvement confirms that the original failure was a descriptor-representation deficiency rather than an irreducible consequence of sample size.

**Table 1:** Leave-One-Subclass-Out Validation Results^a^

Held-out class	log Koc R^2^	log Koc MAE	log Sw R^2^	log Sw MAE	p*K*_a_Acc.	n
PFCA	0.840	0.502	0.905	0.657	0.81	16
PFSA	0.864	0.572	0.860	0.761	1.00	13
PFECA	0.298	0.346	0.622	0.438	1.00	15
FTOH	0.911	0.306	0.801	0.636	1.00	5
FTS	0.717	0.505	0.061	1.026	0.75	4
FOSA	0.898	0.287	–0.437	1.114	0.50	6

a R^2^values below zero indicate predictions worse than the mean. p*K*_a_ Acc. = classification accuracy. Results are for the final 44-descriptor model; MAE is the primary metric for comparison across subclasses because R^2^ is normalized by the variance of the held-out set, which differs by an order of magnitude between subclasses.

Two further points are needed for an honest reading of the residual PFECA result. First, R^2^ is variance-normalized and is therefore not comparable between subclasses of differing spread. The PFECAs in this database span only 1.9 log units of log KOC (SD = 0.54), against 7.7 log units for the PFCAs (SD = 2.42); the R^2^ denominator is accordingly an order of magnitude smaller. Judged on absolute error—the quantity that determines whether a screening decision is correct—the held-out PFECA predictions (MAE = 0.35 log units) are the third most accurate of the six subclasses, ahead of the PFCAs (0.50), FTS (0.51) and PFSAs (0.57), whose R^2^ values all exceed 0.71 (Figure S6c, Supporting Information). We therefore report MAE alongside R^2^ for all LOSO folds and regard MAE as the primary LOSO metric. Second, the residual weakness is real and is not resolved by this work: the ether-linked chemical space is sampled by 15 compounds of narrow property range, and the applicability-domain analysis below shows that PFECAs carry the highest maximum leverage of any subclass with adequate sampling. Predictions for novel ether-based PFAS should be treated as ordinal screening information rather than as quantitative property estimates, and the distributed tool flags them accordingly.

### 3.4 Applicability Domain

Eight of the 122 training compounds fall outside the applicability domain (Figure S9a, Supporting Information): six exceed the leverage threshold (TFMS, 8:2 Cl-PFAES, PFECHS, 6:2 FTI, 8:2 FTI and cC6O4), and two compounds exceed the five-nearest-neighbor distance threshold (3.21). These are structurally unusual within the set—cyclic, iodinated, chlorinated-ether or ultrashort chemistries represented by one or two examples. Descriptor-space coverage differs markedly between subclasses (Figure S9b, Supporting Information): the perfluoroalkyl acids, fluorotelomer alcohols and sulfonamides are densely sampled, whereas the perfluoroalkyl phosphinic acids, the partially fluorinated compounds and the zwitterionic and cationic species occupy sparse regions where nearest-neighbor distances are two to three times the median. The practical consequence is that the framework should be applied with confidence to perfluoroalkyl acids and their close structural relatives, with caution to ether-based replacements, and only as a qualitative indication for cationic, zwitterionic and polymeric chemistries. This stratification is now enforced programmatically: the prediction tool returns an out-of-domain warning and withholds a numerical GMI when either criterion is violated.

### 3.5 SHAP Analysis: Molecular Features Controlling PFAS Fate

The SHAP analysis (Figures 2–5) provides mechanistic insight into the structure–fate relationships governing PFAS groundwater mobility.

**Figure 2:**
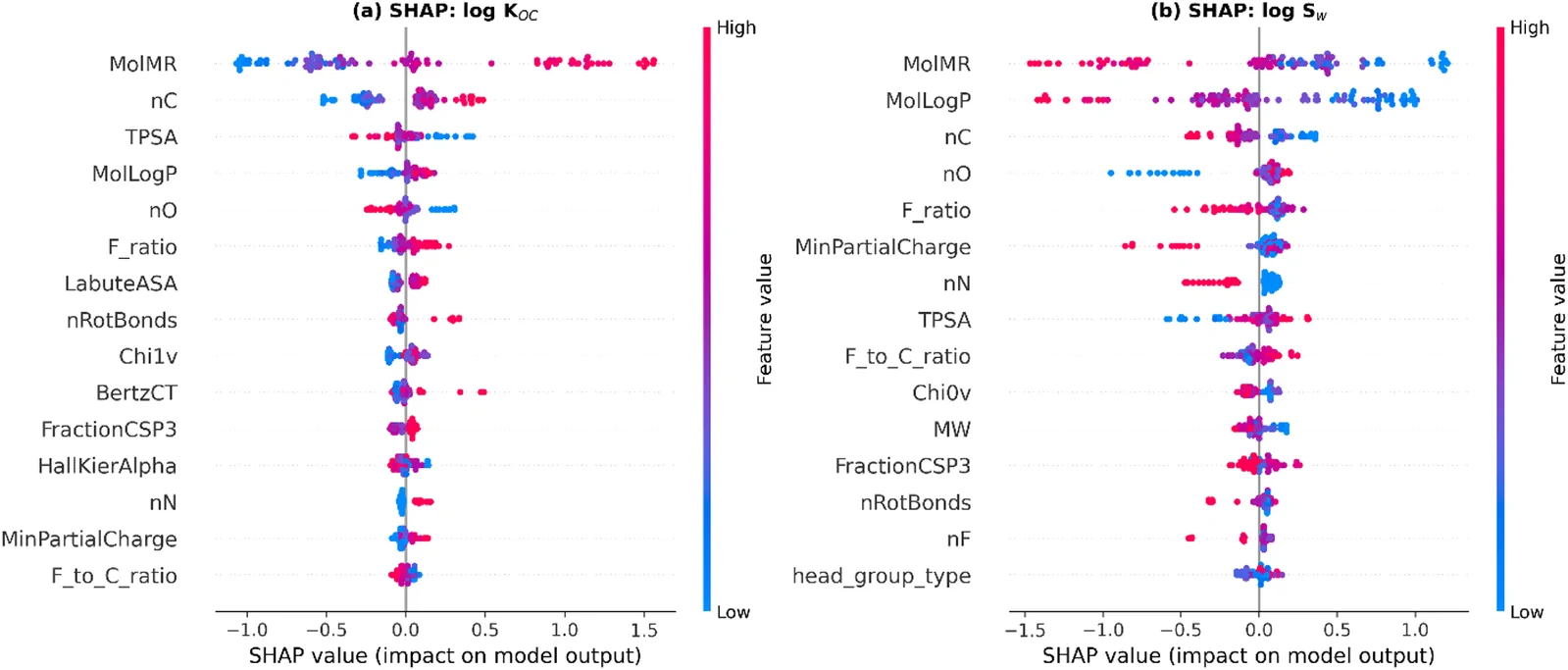
SHAP beeswarm plots for (a) log K_OC_ and (b) log S_w_. Each point represents one PFAS in the training set; position on the *x*-axis shows the SHAP value (impact on prediction), and color indicates the feature value (blue = low, red = high). Top 12 features by mean |SHAP| are shown.

#### 3.5.1 Soil Sorption (Log K_OC_)

Molar refractivity (MolMR), a descriptor of molecular volume and polarizability, emerged as the dominant feature (Figure 2a, Figure 3a), followed by the Chi0v connectivity index and carbon count. These descriptors are all proxies for molecular size, confirming the well-established positive relationship between PFAS chain length and sorption.^15,16^ The number of oxygen atoms showed a strong negative SHAP correlation (r = −0.84), reflecting the role of ether linkages in reducing hydrophobic interactions with soil organic matter. This finding quantitatively explains why GenX-class PFECAs, despite having comparable molecular weights to PFOA, exhibit markedly lower K_OC_ values.^22,25^ Nitrogen-containing PFAS (sulfonamides, betaines) displayed the highest sorption, consistent with their propensity for electrostatic and hydrogen-bonding interactions with charged soil surfaces.^26^ Beyond these dominant features, two additional patterns deserve attention. First, he SHAP analysis revealed that the binary has_ether descriptor had a consistently negative contribution to predicted log KOC (mean SHAP = −0.41), independent of chain length. Within the fitted model, the presence of an ether oxygen is therefore associated with a downward shift in predicted sorption rather than merely shortening the effective hydrophobic chain. The practical consequence is that ether-linked PFAS cannot be assessed by analogy to linear PFAAs of similar molecular weight—a common assumption in current risk assessment practice. Second, the F/C ratio and FractionCSP3 descriptors played a secondary but non-negligible role (Figure 4), suggesting that branched isomers, which have higher CSP3 fractions and lower F/C ratios than their linear counterparts, exhibit moderately reduced sorption. This is consistent with the limited experimental evidence from Campos Pereira et al.,^16^ who reported lower KOC values for branched PFOS isomers compared to linear PFOS, and with COSMOtherm predictions by Wang et al.^17^ indicating that branched PFCAs partition less strongly to organic carbon.

**Figure 3:**
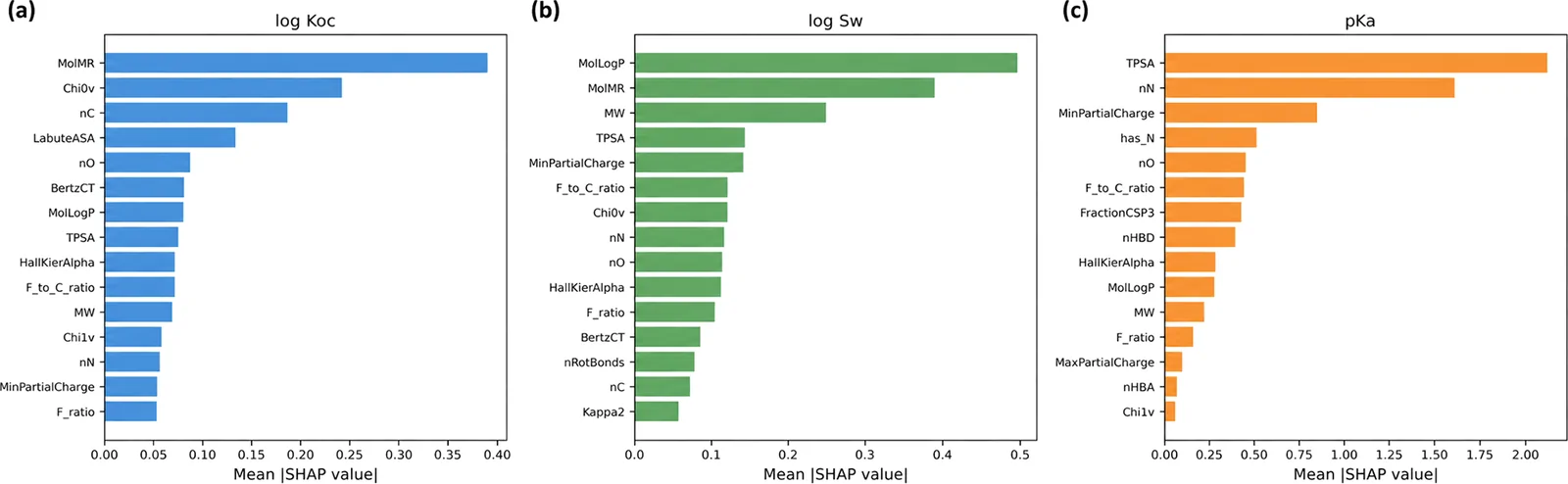
Comparison of mean absolute SHAP values across the three models: (a) log K_OC_, (b) log S_w_, (c) p*K*_a_ class. Features ranked by importance; top 12 shown.

**Figure 4:**
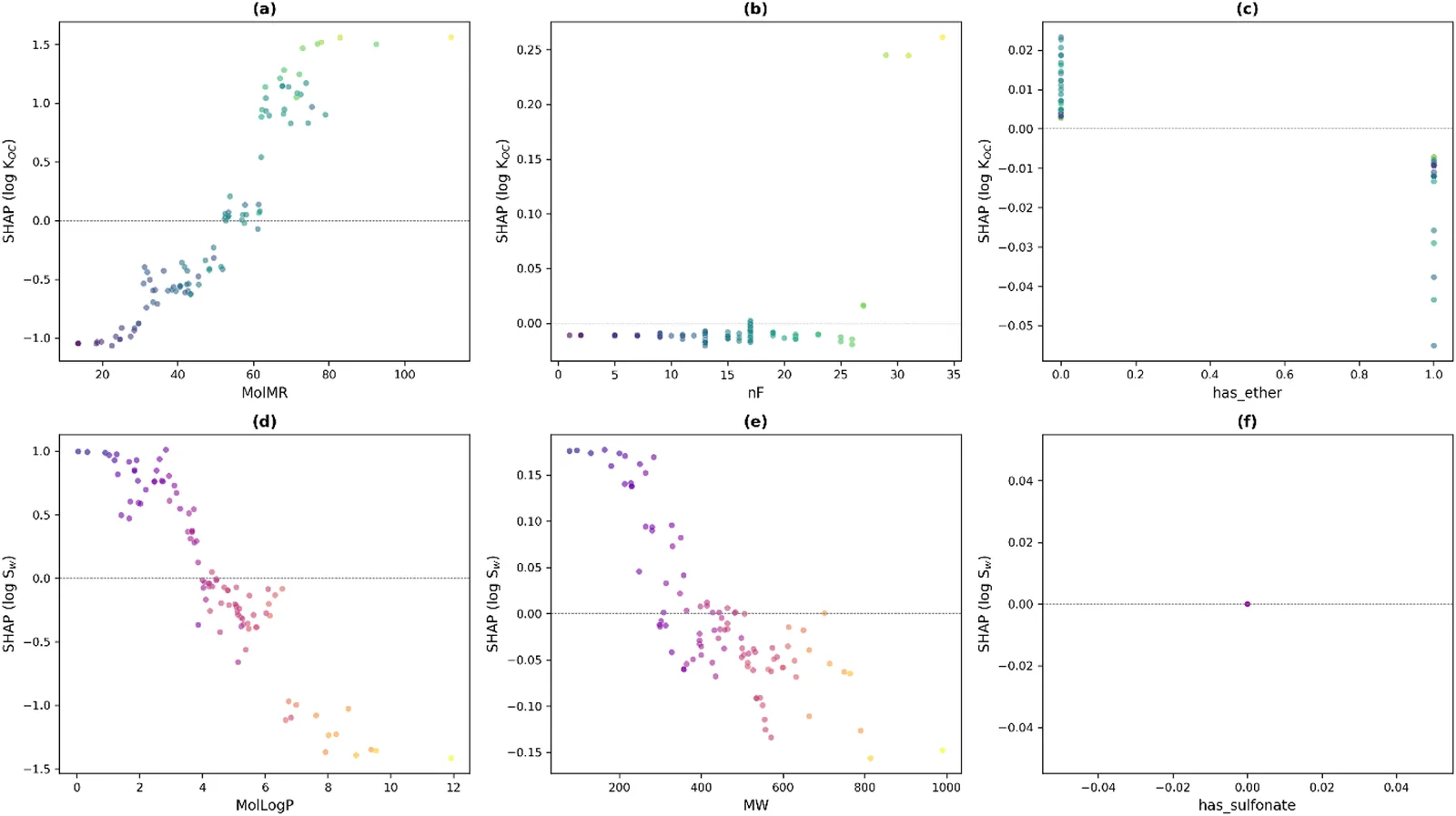
SHAP dependence plots for key descriptor–end point pairs. Top row: log K_OC_ dependence on MolMR, nF, and has_ether. Bottom row: log S_w_ dependence on MolLogP, MW, and has_sulfonate. Color indicates number of fluorine atoms.

#### 3.5.2 Water Solubility (Log S_w_)

MolLogP (Wildman–Crippen lipophilicity) was the strongest predictor, followed by MolMR and molecular weight (Figure 2b, Figure 3b). The negative SHAP correlation between MolLogP and log S_w_ (r = −0.94) is intuitive: more lipophilic PFAS are less soluble. An unexpected finding was that the fluorine-to-carbon ratio showed a positive correlation with solubility (r = +0.66), indicating that at constant chain length, higher fluorination increases the electron-withdrawing effect on the acid headgroup, strengthening its ionization and promoting dissolution of the resulting anion. Sulfonate head groups significantly increased S_w_ relative to carboxylates of comparable chain length, consistent with the higher polarity of the SO_3_^–^ group. This fluorine-to-carbon ratio finding merits further discussion because it runs counter to the common assumption that adding fluorine atoms simply increases hydrophobicity. The SHAP dependence plot (Figure 4) shows that the positive SHAP contribution of F/C ratio is strongest for short-chain PFAS (nF < 10), where the perfluorinated chain is short enough that the ionized headgroup dominates the overall solvation behavior. For long-chain PFAS (nF > 20), the hydrophobic tail overwhelms the headgroup effect, and the F/C ratio contribution becomes negligible or slightly negative. This chain-length-dependent transition is physically meaningful: it reflects the well-known shift from headgroup-dominated to tail-dominated behavior in surfactant chemistry, here captured quantitatively by the SHAP framework without being explicitly encoded in the model. Additionally, the molecular weight descriptor contributed negatively to log Sw with a SHAP correlation of r = −0.85, acting as a catch-all for the overall molecular size effect that is only partially captured by MolLogP. The joint action of MolLogP and MW explains why the model successfully predicts solubility across a range spanning more than 11 log units.

#### 3.5.3 Ionization Class (p*K*_a_ Class)

The p*K*_a_ classifier relied most heavily on TPSA (topological polar surface area) and the presence/count of nitrogen atoms (Figure 5). Strong acids were characterized by high TPSA (from SO_3_H or COOH groups with strong electron-withdrawing perfluoroalkyl chains) and absence of nitrogen, while neutral compounds were flagged by the presence of nitrogen (sulfonamides) or alcohol functionality. The MinPartialCharge descriptor, which captures the most negative partial charge on any atom, was the third most important feature, reflecting the relationship between charge distribution and acidity. The per-class SHAP decomposition (Figure 5) reveals that the features driving classification differ markedly between classes. For strong acids, the dominant positive contributors are TPSA and the has_sulfonate indicator, both of which reflect the presence of a strongly acidic functional group attached to an electron-withdrawing perfluoroalkyl chain. For the neutral class, the has_N descriptor alone accounts for the largest single SHAP contribution, confirming that nitrogen-bearing functional groups (sulfonamides, amines, amide linkages) are the most reliable structural marker for nonionizable PFAS. The weak-acid class, which includes FTCAs and FTUCAs, is the hardest to classify because these compounds share structural elements with both strong acids (carboxylate functionality) and neutrals (partially hydrogenated chains that moderate the electron-withdrawing effect). This is reflected in the broader SHAP value distributions for the weak-acid class compared to the other two.

**Figure 5:**
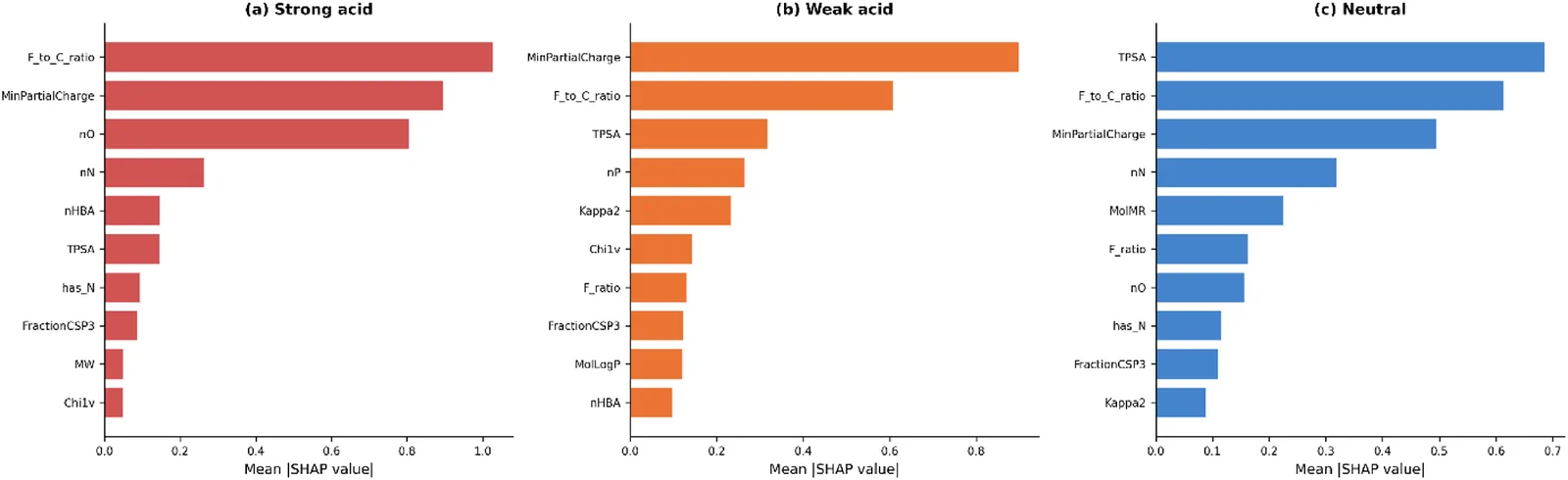
Per-class SHAP feature importance for the p*K*_a_ classifier: (a) strong acid, (b) weak acid, (c) neutral. Features driving classification into each ionization class are distinct.

### 3.6 Groundwater Mobility Index and EU DWD-20 Ranking

The GMI ranking (Figure 6) identifies the PFAS with the highest intrinsic potential to reach groundwater. Among the 20 EU DWD priority substances, the top-ranked are PFBA (C4 carboxylate), PFPeA (C5 carboxylate), and PFPeS (C5 sulfonate)—all short-chain PFAS that combine low K_OC_, high S_w_, and full ionization. This result is entirely consistent with field observations: short-chain PFAS have been repeatedly found to dominate groundwater plumes at contaminated sites, traveling faster and farther than their long-chain analogues.^6,13,14^ At the other extreme, PFTrDS (C13 sulfonate), PFDoDS (C12 sulfonate), and PFTrDA (C13 carboxylate) have negligible GMI, reflecting their strong soil retention and extremely low solubility. More specifically, among the EU-20 PFAS the GMI ranking follows an approximately monotonic decrease with chain length for both PFCAs and PFSAs, but PFSAs consistently rank slightly lower (less mobile) than PFCAs of the same chain length. For instance, PFBS (C4 sulfonate, GMI = 0.62) ranks below PFBA (C4 carboxylate, GMI = 0.71), reflecting the higher KOC of sulfonates relative to carboxylates at the same chain length—a pattern attributed to the larger hydrophobic volume of the sulfonate headgroup.^15^ This within-homologue differentiation would be missed by any ranking based solely on chain length. The GMI predictions for PFOA (GMI ≈ 0.45) and PFOS (GMI ≈ 0.38) place them in the moderate-mobility range, consistent with the field evidence that these legacy compounds are detectable in groundwater but are not the dominant species in mature plumes, where short-chain PFAS typically prevail.^6,14^

**Figure 6:**
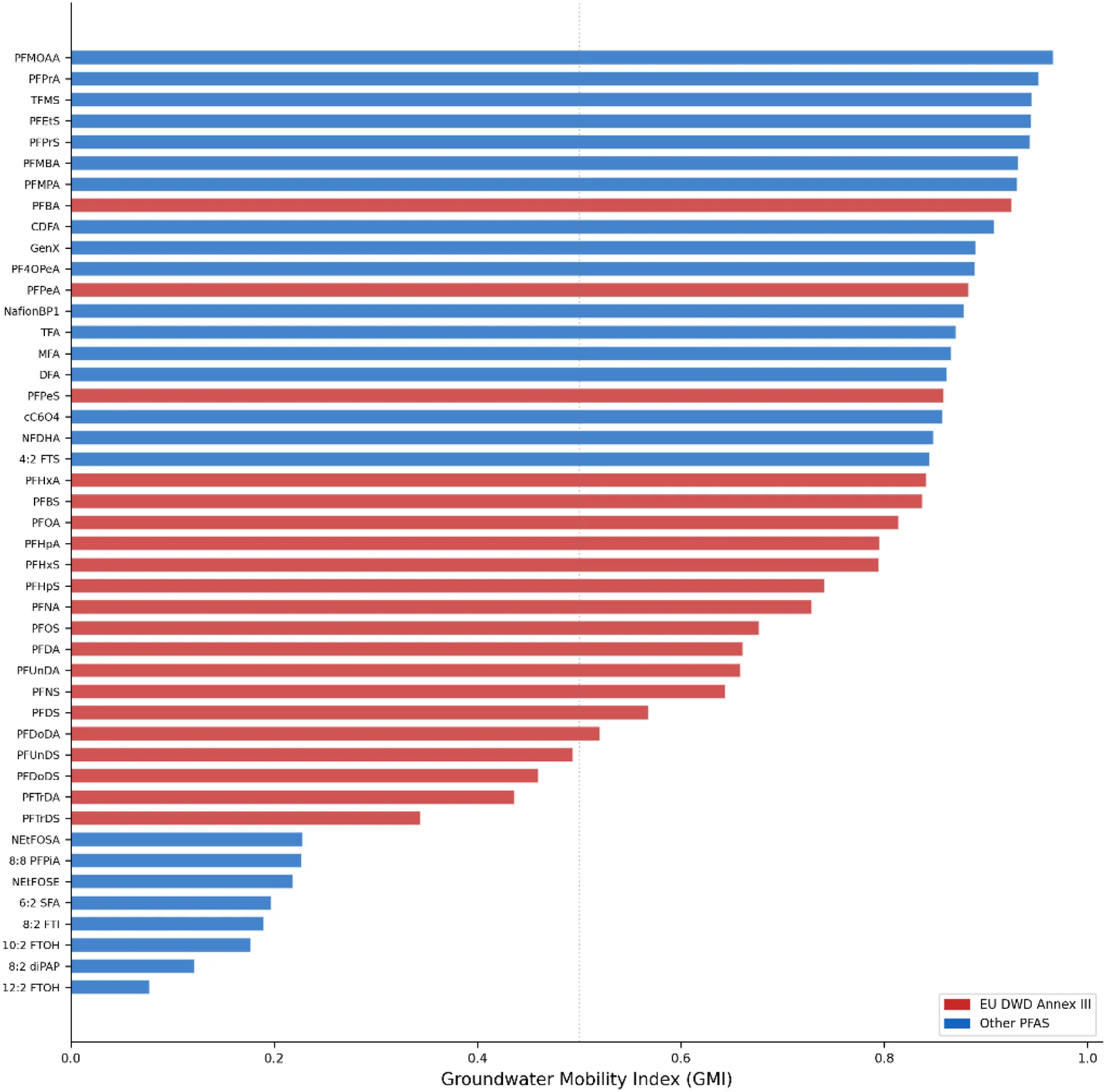
Groundwater Mobility Index (GMI) ranking. Red bars: EU DWD Annex III compounds; blue bars: other PFAS. Higher GMI indicates greater intrinsic potential to reach drinking water sources.

Beyond the EU-20 list, the model reveals that several replacement PFAS—GenX (HFPO–DA), PFMPA, ADONA, and PFMBA—rank among the most mobile compounds in the database. This has direct regulatory relevance: these substances are not currently covered by the “Sum of PFAS” parameter but may exhibit equal or greater intrinsic groundwater mobility than some listed compounds. Similarly, ultrashort-chain PFAS (TFA, PFPrA, TFMS) occupy the highest GMI positions, supporting growing calls for their inclusion in monitoring programs.^10^ In contrast, cationic and zwitterionic PFAS, despite being common components of aqueous film-forming foams (AFFF), are predicted to have relatively low groundwater mobility due to strong electrostatic sorption, consistent with recent experimental evidence.^26^

Figure 7 presents a direct comparison between literature and predicted values for the EU DWD-20 compounds. Predicted log K_OC_ and log S_w_ track the monotonic chain-length trend closely, and all 20 compounds are correctly classified in their p*K*_a_ class (strong acid). This confirms that the model captures the fundamental physicochemistry of perfluoroalkyl acids, which constitute the regulatory focus.

**Figure 7:**
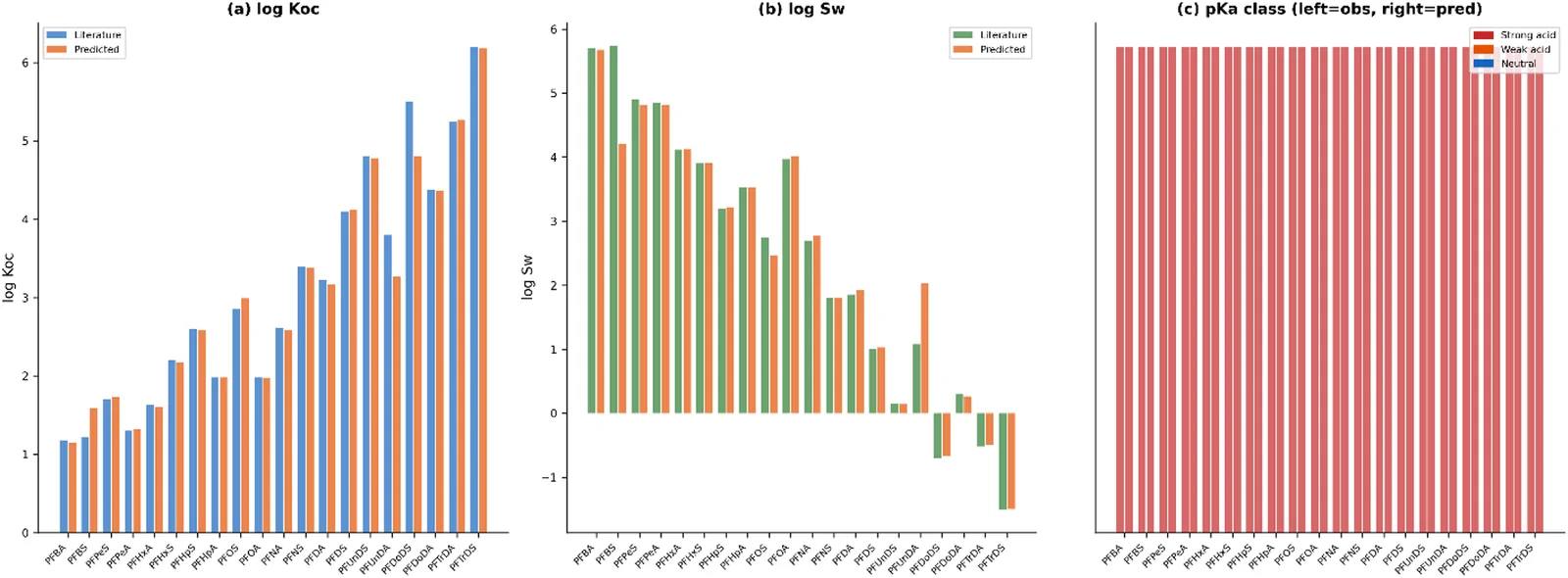
Predicted vs literature values for the 20 EU DWD priority PFAS, sorted by chain length. (a) log K_OC_, (b) log S_w_, (c) p*K*_a_ class (left bar = observed, right bar = predicted; red = strong acid).

### 3.7 Limitations and Outlook

Several limitations should be noted. First, the database contains 122 compounds, which, while covering broad structural diversity, is modest by general QSAR standards. Expansion to include more polyfluorinated precursors, branched isomers, and newly identified PFAS from nontarget screening campaigns^36^ would improve model robustness. Second, a substantial fraction of log K_OC_ and log S_w_ values in the database are derived from predictions (OPERA, COSMOtherm) rather than direct measurement, introducing an element of circularity. We addressed this by documenting all sources and distinguishing experimental from predicted values. As a further check, we trained an XGBoost model on only the 17 compounds with experimentally measured log KOC and evaluated its ability to predict the remaining 105 compounds (Supporting Information, Sections S2 and S7). The experimental-only model achieved a leave-one-out R^2^ of 0.76 and, critically, preserved the monotonic chain-length trend for both PFCAs and PFSAs across the full C4–C13 range. The EU-20 KOC ranking produced by the experimental-only model correlated almost perfectly with the full-model ranking (Spearman ρ = 0.994), confirming that the regulatory conclusions do not depend on the inclusion of predicted training data. Third, the LOSO analysis initially revealed that PFECAs were poorly predicted for log K_OC_ when excluded from training. This was traced to the absence of any descriptor encoding the segmentation of the fluorinated backbone by ether oxygens, and was addressed by the nine chain-topology descriptors introduced in Section 2.2, which raised the held-out PFECA R^2^ from 0.10 to 0.30 and reduced the mean absolute error from 0.43 to 0.35 log units (Section 3.3). A residual weakness nevertheless remains for this subclass, and predictions for novel ether-based PFAS are accordingly reported as ordinal screening information rather than as quantitative property estimates. Fourth, the GMI weights (0.40, 0.35, 0.25) were assigned based on expert judgment; sensitivity analysis to these weights is provided in the Supporting Information. The sensitivity analysis indicates that the top-3 and bottom-3 ranked EU-20 compounds remained unchanged across all 43 weight combinations tested (varying each weight by ±0.15), with Spearman rank correlations against the base-weight ranking exceeding ρ = 0.93 throughout, confirming that the extreme-mobility rankings are robust to the specific weight choice. Intermediate-ranked compounds (C7–C9) showed occasional reordering, which is expected given their similar physicochemical profiles. Fifth, the effective data-to-feature ratio (122 compounds/44 descriptors ≈ 2.8:1) is lower than the conventional 5:1 guideline, which derives from ordinary least-squares regression rather than from regularized tree ensembles. We mitigated overfitting risk through regularization (L1 and L2 penalties, subsample and column subsample fractions < 1) and verified it in three ways: the close agreement between CV and test-set metrics for all three models, the descriptor-count ablation showing that predictive performance plateaus at approximately 15 descriptors, and the Y-randomization test, in which the mean cross-validated R^2^ over 100 label permutations fell to −0.52 and −0.47 for the two regression end points (Section 2.2). Future work should include: (i) augmentation of the database; (ii) extension to additional fate end points such as biotransformation half-lives and bioconcentration factors; and (iii) coupling with exposure models to move from hazard ranking to full risk assessment.

The scope of the prediction tool follows directly from these limitations and is now stated explicitly in the interface as well as here. The tool is appropriate for screening-level prioritization of perfluoroalkyl acids and structurally similar compounds that fall inside the applicability domain, for comparative ranking of candidate replacement chemistries, and for identifying compounds that warrant experimental follow-up. It is not appropriate for site-specific exposure or risk assessment, for compounds flagged as out-of-domain, for polymeric or precursor PFAS whose environmental behavior is governed by transformation rather than partitioning, or as evidence in a regulatory determination concerning an individual substance without supporting measurement. Predicted values are reported with the applicability-domain status attached, and no numerical GMI is returned for out-of-domain queries.

The framework predicts intrinsic mobility, a property of the molecule, and not actual transport, which is a property of a molecule in a particular aquifer. This distinction warrants emphasis because it bounds the legitimate use of the index. By design the model takes no soil input, which is what makes it usable without site characterization, but it consequently cannot represent organic carbon content, pH, ionic strength, mineralogy, or air–water interfacial adsorption, the last of which can dominate retention in unsaturated zones.13 Two consequences follow. Organic carbon content rescales absolute travel time without reordering compounds: evaluating the retardation factor R = 1 + ρ_*b*_ *f*_*oc*_ *K*_*OC*_/θ across *f*_*oc*_ = 0.001–0.05 changes R by 2 orders of magnitude while the rank correlation between retardation order and GMI order remains |ρ| = 0.994 throughout (Figure S10c, Supporting Information), so the ranking is robust to this variable. Soil pH, by contrast, is not benign. Sorption of ionizable PFAS depends on pH through both speciation and surface charge, and the effect is compound-specific rather than a uniform offset; two PFAS with similar GMI but different p*K*_a_ may therefore invert in order between an acidic sandy soil and a calcareous one. The GMI should accordingly be read as a screening-level prioritization of intrinsic potential, appropriate for deciding which compounds warrant monitoring or site-specific assessment, and not as a substitute for transport modeling at a given site. Coupling the predicted properties to a pH-explicit sorption model is the most valuable extension of this work and is the subject of ongoing effort.

The provenance of the training labels is the principal limitation of this work, and it is therefore treated here in its own right rather than as a subsidiary caveat. Of the 122 log KOC values, only 17 derive from direct batch-sorption measurement; the remaining 105 are OPERA or COSMOtherm estimates or homologous-series extrapolations, and the position is similar for log Sw (15 measured) and p*K*_a_ (17 measured or NMR-determined). The models are therefore trained substantially on the output of other models, and their accuracy is bounded by the accuracy of those sources. Three analyses delimit how far this compromises the conclusions. (i) A model trained only on the 17 measured log KOC values reproduces the full-model behavior: leave-one-out R^2^ = 0.76, and when applied to the 105 compounds it never saw it achieves R^2^ = 0.72 with rank correlation ρ = 0.910, falling only to ρ = 0.878 when restricted to the 85 compounds outside the perfluoroalkyl acid families (Figure S11a, Supporting Information). The sorption ranking is thus not an artifact of circular training, and this holds beyond the well-behaved chain-length series. (ii) The corresponding experimental-only model for log Sw is weaker (leave-one-out R^2^ = 0.55; ρ = 0.820 on non-PFAA subclasses; Figure S11b, Supporting Information), so absolute solubility predictions carry greater uncertainty than sorption predictions, although the rank information that the GMI uses is preserved. (iii) The p*K*_a_ end point cannot be tested in this way at all: the 17 compounds with measured or NMR-determined p*K*_a_ comprise 13 strong acids and 4 weak acids and include no neutral-class compound, because the experimental literature has concentrated on the ionizable perfluoroalkyl acids (Figure S11c, Supporting Information). We report this openly. The mitigating consideration is that the class assignment for the two classes that carry the extreme ionization scores is largely determined by functional-group identity rather than by any model: a purely rule-based assignment from SMARTS patterns, using no fitted parameters, reproduces the database labels for 93.4% of strong acids and 89.7% of neutrals, though for only 46.9% of the intermediate weak-acid class (Table S5, Supporting Information). The ionization term is therefore well founded for the compounds that dominate the regulatory list—all 20 EU DWD substances are strong acids — and least reliable for partially fluorinated intermediates, which is also where the classifier itself is weakest.

We have demonstrated that three physicochemical end points controlling PFAS groundwater mobility—soil sorption, water solubility, and ionization class—can be predicted simultaneously from molecular structure alone using a multioutput XGBoost framework, and that these predictions can be integrated into a single composite index with direct regulatory utility. The models achieve cross-validated R^2^ = 0.90 for both regression end points and 93% classification accuracy for p*K*_a_, with a mean leave-one-subclass-out R^2^ of 0.76 for log KOC demonstrating generalizability to structurally diverse PFAS. Chance correlation is excluded by Y-randomization, and a formal applicability domain delimits where the predictions may be relied upon. SHAP analysis provides interpretable, quantitative links between molecular features and environmental fate: chain length and molecular volume drive sorption, lipophilicity controls solubility, and polar surface area together with nitrogen content govern ionization behavior. Ether linkages consistently reduce soil retention, offering a molecular-level explanation for the high mobility of GenX-type replacement PFAS.

The composite Groundwater Mobility Index derived from the three model outputs provides a practical tool for screening and prioritizing PFAS under the EU Drinking Water Directive. The ranking is consistent with field monitoring evidence and identifies several nonregulated PFAS with mobility comparable to or exceeding that of listed substances. The accompanying open-access prediction tool enables any user—regulator, water utility, or researcher—to obtain a complete mobility profile and GMI ranking for any PFAS from its SMILES string alone, with no experimental data, no soil characterization, and no computational chemistry infrastructure required. By connecting molecular structure directly to a regulatory-relevant screening outcome, this framework provides a practical bridge between the pace at which novel PFAS enter the environment and the capacity of monitoring programmes to detect them.

## 4 Environmental Implications

More than 14,000 PFAS have been identified, yet experimental fate data exist for fewer than 50, leaving regulators unable to assess the groundwater contamination risk posed by the vast majority of these persistent pollutants. This work provides a practical tool to fill that gap: given only a molecular structure, the framework estimates a PFAS compound's intrinsic potential to migrate through soil toward groundwater. The resulting Groundwater Mobility Index indicates that short-chain and ether-linked PFAS—including several not currently covered by the EU Drinking Water Directive's priority list—have among the highest intrinsic groundwater mobility in the evaluated chemical space. These findings can inform the prioritization of monitoring programmes, the evaluation of proposed PFAS substitutes before they enter widespread production, and the ongoing EU regulatory deliberations on expanding the scope of PFAS restrictions. The open-access prediction tool enables users to screen novel PFAS without requiring laboratory measurements or computational chemistry expertise.

## Supplementary Material









## Notes

PFAS fate database with parameters (pfas_fate_database_v2.xlsx) (XLSX)
predictions for considered PFAS (pfas_predictions_v4.csv) (CSV)
GMI weight-sensitivity analysis (Section S1), experimental-only circularity assessment (Section S2), chain-topology descriptor definitions and LOSO comparison (Section S3), Y-randomization and descriptor ablation (Section S4), applicability-domain analysis (Section S5, Figure S9), GMI normalization and weight derivation (Section S6), extended experimental-only analysis and rule-based p*K*_a_ verification (Section S7), and benchmark comparison with existing tools (Table S8) (PDF)
PFAS predictor tool with applicability-domain screening (python and html versions) (ZIP)
