# Supplementary material for: Structure-Based Prediction and Ranking of PFAS Groundwater Mobility for EU Drinking Water Regulation
Source: Environ Sci Technol. 2026 Sep 29;60(40):29077–88. doi: 10.1021/acs.est.6c11513 (PMC13674058; doi:10.1021/acs.est.6c11513)
Supplement: Supplementary file 1 [file es6c11513_si_003.pdf]

Supporting Information

Structure-Based Prediction and Ranking of PFAS Groundwater Mobility for EU Drinking Water Regulation

Sergio de-la-Huerta,[a] Ozge Ozkilinc,[a,b] Valentín Diez-Cabanes,[a,b] María Antonieta Escobedo,[a,b] Pedro A. Marcos,[c] Alfredo Bol,[c] Mert Atilhan,[d] Rocio Barros,[b] Santiago Aparicio[a,b*]

[a] *Department of Chemistry, University of Burgos, 09001 Burgos, Spain*
[b] *ICCRAM (International Research Centre in Critical Raw Materials and Advanced Industrial Technologies), 09001 Burgos, Spain*
[c] *Department of Physics, University of Burgos, 09001 Burgos, Spain*
[d] *Department of Chemical and Paper Engineering, Western Michigan University, Kalamazoo MI 49008-5462, USA*

* *Corresponding author: sapar@ubu.es*

S1. GMI WEIGHT SENSITIVITY ANALYSIS

S1.1. Methodology

The Groundwater Mobility Index (GMI) combines three predicted endpoints using the weighted formula:

$$GMI = w_{KOC} \times norm(-\log K_{OC}) + w_{Sw} \times norm(\log S_w) + w_{ion} \times I_{ion}$$

where the base weights are w_{KOC} = 0.40, w_{Sw} = 0.35, and w_{ion} = 0.25. To evaluate the sensitivity of the PFAS mobility ranking to these weight assignments, we conducted a systematic perturbation analysis. Each weight was varied in increments of 0.05 over a range of ±0.15 from the base value, subject to two constraints: (i) the three weights must sum to 1.00, and (ii) each individual weight must remain ≥ 0.05 to prevent any endpoint from being effectively excluded. This procedure generated 43 distinct weight combinations spanning w_{KOC} = 0.25–0.55, w_{Sw} = 0.20–0.50, and w_{ion} = 0.10–0.55.

For each weight combination, the GMI was recalculated for all 122 PFAS compounds. Ranking stability was assessed through three metrics: (i) Spearman rank correlation (ρ) between each alternative ranking and the base-weight ranking, computed over all 122 compounds; (ii) positional stability of each EU DWD-20 compound, summarized as the median, mean, minimum, and maximum rank observed across all weight combinations; and (iii) set stability of the top-3 and bottom-3 most mobile EU-20 compounds.

Additionally, six extreme weight scenarios were examined to probe the boundaries of ranking behavior: a sorption-dominated scenario (0.70/0.15/0.15), a solubility-dominated scenario (0.15/0.70/0.15), an ionization-dominated scenario (0.15/0.15/0.70), equal weights (0.33/0.34/0.33), and a scenario that entirely excludes ionization (0.55/0.45/0.00).

S1.2. Overall Ranking Robustness

The Spearman rank correlation coefficient between the base-weight ranking and each alternative ranking ranged from ρ = 0.932 to 1.000, with a mean of 0.985 and a median of 0.989 (Figure S2). All 43 weight combinations yielded ρ > 0.90, and 40 out of 43 (93%) exceeded ρ = 0.95. This indicates that the overall ordering of PFAS by groundwater mobility is highly insensitive to the specific weight choice within the tested range. The ranking is predominantly determined by the underlying physicochemical property predictions rather than by the weighting scheme.

The slight reductions in ρ observed at extreme weight combinations (e.g., w_{ion} = 0.55) reflect reordering among compounds with similar GMI values in the intermediate-mobility range. Compounds at the extremes of the ranking—the most mobile and least mobile—are essentially invariant to weight perturbation, as discussed below.

S1.3. EU DWD-20 Compound Ranking Stability

Table S1 presents the complete ranking statistics for the 20 EU DWD Annex III compounds across all 43 weight combinations. The results demonstrate remarkable stability:

(i) **18 of 20 compounds (90%)** occupy the exact same rank in every single weight combination tested. Their rank range is zero, meaning their position in the mobility ordering is entirely independent of the weight choice.

(ii) The only two compounds showing any rank variation are **PFBS/PFPeS** (which swap between positions 3 and 4) and **PFHxS/PFHpA** (which swap between positions 7 and 8). In both cases, the swapping compounds have nearly identical base GMI values (PFBS: 0.867, PFPeS: 0.865; PFHxS: 0.801, PFHpA: 0.798), making rank interchange at this precision physically insignificant.

(iii) **The top-3 most mobile EU-20 PFAS** (PFBA, PFPeA, and PFBS/PFPeS) remained unchanged in 79% of weight combinations and were always drawn from the same set of four short-chain compounds (PFBA, PFPeA, PFBS, PFPeS).

(iv) **The bottom-3 least mobile EU-20 PFAS** (PFUnDS, PFTrDA, PFTrDS) remained identical in 100% of weight combinations, without exception.

Figure S1 visualizes the rank distribution for each EU-20 compound as a boxplot. The absence of whiskers for most compounds confirms the zero-variability finding. The two pairs of swapping compounds are visible as the only boxes with nonzero height.

Table S1. Ranking stability of EU DWD Annex III PFAS across 43 weight combinations of the Groundwater Mobility Index. Median rank, mean rank, minimum and maximum rank, rank range, and base GMI value (w_{KOC} = 0.40, w_{Sw} = 0.35, w_{ion} = 0.25) are reported for each compound.

Compound	Class	Chain	Median rank	Mean rank	Min rank	Max rank	Rank range	Base GMI
**PFBA**	PFCA	C4	1	1	1	1	0	0.930
**PFPeA**	PFCA	C5	2	2	2	2	0	0.888
**PFBS**	PFSA	C4	3	3.2	3	4	1	0.867
**PFPeS**	PFSA	C5	4	3.8	3	4	1	0.865
**PFHxA**	PFCA	C6	5	5	5	5	0	0.842
**PFOA**	PFCA	C8	6	6	6	6	0	0.817
**PFHxS**	PFSA	C6	7	7.3	7	8	1	0.801
**PFHpA**	PFCA	C7	8	7.7	7	8	1	0.798
**PFHpS**	PFSA	C7	9	9	9	9	0	0.748
**PFNA**	PFCA	C9	10	10	10	10	0	0.730
**PFOS**	PFSA	C8	11	11	11	11	0	0.713
**PFDA**	PFCA	C10	12	12	12	12	0	0.664
**PFUnDA**	PFCA	C11	13	13	13	13	0	0.651

**PFNS**	PFSA	C9	14	14	14	14	0	0.646
**PFDS**	PFSA	C10	15	15	15	15	0	0.571
**PFDoDA**	PFCA	C12	16	16	16	16	0	0.526
**PFDoDS**	PFSA	C12	17	17	17	17	0	0.517
**PFUnDS**	PFSA	C11	18	18	18	18	0	0.499
**PFTrDA**	PFCA	C13	19	19	19	19	0	0.447
**PFTrDS**	PFSA	C13	20	20	20	20	0	0.346

Red shading: top-5 most mobile. Green shading: bottom-5 least mobile. Rank range = 0 indicates the compound occupies the same position under all 43 weight combinations.

S1.4. Extreme Weight Scenarios

Table S2 presents the top-5 EU-20 PFAS ranking under six extreme weight scenarios, including cases where a single endpoint is given dominant weight (70%) and a scenario that entirely excludes the ionization component.

Table S2. Top-5 EU DWD-20 compounds by GMI under extreme weight scenarios.

Scenario	$w_{□oc}$	w□□	$w_{ioₙ}$	Rank 1	Rank 2	Rank 3	Rank 4	Rank 5
**Base**	0.40	0.35	0.25	**PFBA**	**PFPeA**	**PFBS**	**PFPeS**	**PFHxA**
**Sorption-dominated**	0.70	0.15	0.15	**PFBA**	**PFPeA**	**PFBS**	**PFPeS**	**PFHxA**
**Solubility-dominated**	0.15	0.70	0.15	**PFBA**	**PFPeA**	**PFPeS**	**PFBS**	**PFHxA**
**Ionization-dominated**	0.15	0.15	0.70	**PFBA**	**PFPeA**	**PFBS**	**PFPeS**	**PFHxA**
**Equal weights**	0.33	0.34	0.33	**PFBA**	**PFPeA**	**PFBS**	**PFPeS**	**PFHxA**
**No ionization**	0.55	0.45	0.00	**PFBA**	**PFPeA**	**PFBS**	**PFPeS**	**PFHxA**

The most striking finding is the near-total invariance of the top-5 ranking: PFBA and PFPeA occupy the first and second positions in every scenario tested, including the extreme cases. The only variation occurs in positions 3–4, where PFBS and PFPeS occasionally swap—a difference that is physically insignificant given their nearly identical GMI values. PFHxA consistently holds position 5. This robustness arises because PFBA and PFPeA have the lowest K_{OC}, the highest S_w, and full ionization simultaneously—they rank at the top on all three component scores, making their overall ranking independent of how these components are weighted.

Even the scenario that completely excludes ionization (w_{ion} = 0) produces the identical top-5 ranking, confirming that for the EU DWD-20 compounds (which are all strong acids), the ionization component adds redundant rather than discriminating information. This is an important result because the pK_a classification is the model with the lowest accuracy; the analysis shows that the GMI ranking would be unchanged even if pK_a were entirely omitted for this compound set.

S1.5. GMI Value Distributions for Individual Compounds

Figure S3 presents the distribution of GMI values for six selected EU DWD-20 compounds (PFBA, PFPeA, PFOA, PFOS, PFTrDA, PFTrDS) across all 43 weight combinations. The standard deviations are small (0.01–0.04) relative to the GMI differences between compounds, confirming that the weight perturbations produce only minor fluctuations in absolute GMI values. The distributions for the most mobile (PFBA, PFPeA) and least mobile (PFTrDA, PFTrDS) compounds are particularly narrow, reinforcing the conclusion that extreme-ranked compounds are insensitive to weight choice.

The moderate-mobility compounds (PFOA, PFOS) show slightly broader distributions, reflecting the fact that at intermediate positions, the three GMI components contribute more heterogeneously to the overall score. However, even for PFOA and PFOS, the standard deviation (0.02–0.03) is an order of magnitude smaller than the gap separating them from their nearest neighbors in the ranking, ensuring that their positions remain stable.

S1.6. Summary and Implications

The sensitivity analysis leads to three principal conclusions:

- First, the GMI ranking is overwhelmingly robust. The Spearman rank correlation between the base-weight ranking and any alternative weight combination tested exceeds 0.93, and 93% of combinations exceed ρ = 0.95. The ranking is driven by the physicochemical property predictions, not by the weight assignment.
- Second, the compounds at the top and bottom of the EU DWD-20 ranking are effectively invariant. PFBA and PFPeA are always the most mobile; PFTrDA and PFTrDS are always the least mobile. The only weight-sensitive region involves two pairs of compounds with near-identical properties (PFBS/PFPeS and PFHxS/PFHpA).
- Third, the base weights (0.40/0.35/0.25) represent a reasonable choice within a broad equivalence class—any combination within the tested range produces functionally identical

regulatory conclusions. This means that the policy-relevant findings of the study (identification of most and least mobile PFAS, identification of non-regulated mobile compounds) are not contingent on the specific weight values chosen.

S2. Experimental-Only Model: Circularity Assessment

S2.1. Rationale

A potential concern with the log K_{OC} model is that a substantial fraction of the training data (105 of 122 entries) consists of values derived from OPERA, COSMOtherm, or homologous-series extrapolation rather than direct experimental measurement. If the model were simply memorizing patterns within these predicted values, its apparent accuracy could be inflated — in effect, predicting predictions. To assess the severity of this issue, we trained a separate XGBoost model exclusively on the 17 compounds for which experimentally measured log KOC values are available (from batch sorption experiments reported in refs 15, 16, 26, and related studies). These 17 compounds span four subclasses (7 PFCAs, 3 PFSAs, 3 FTOHs, 2 FTS, 1 FOSAA, 1 PFECA) and cover a log KOC range of 1.18–5.25, providing reasonable coverage of the property space despite the small sample size.

S2.2. Methods

Model hyperparameters were adjusted for the small training set: 80 trees, maximum depth 3, learning rate 0.10, subsample 0.90, column subsample 0.70, L1 regularization 0.20, and L2 regularization 2.0. Leave-one-out (LOO) cross-validation was used to evaluate internal accuracy, as k-fold CV is unreliable with $n = 17$. The trained model was then applied to predict log KOC for the remaining 105 non-experimental compounds. The chain-length trend was assessed by predicting log KOC for all 20 EU DWD compounds and checking for monotonicity within the PFCA and PFSA homologous series. Finally, the EU-20 KOC ranking from the experimental-only model was compared to that from the full 122-compound model via the Spearman rank correlation coefficient.

S2.3. Results

The experimental-only model achieved LOO $R^2 = 0.66$, MAE = 0.47 log units, and Pearson $r = 0.82$ (Figure S5a). The lower accuracy relative to the full model (CV $R^2 = 0.90$) is expected given the 7-fold reduction in training size and the loss of structural diversity. Nevertheless, all predictions

fall within ±1.0 log unit of the measured values, and the model correctly ranks the experimental compounds from lowest (PFBA, log KOC = 1.18) to highest (PFTrDA, log KOC = 5.25) sorption.

When applied to the 105 non-experimental compounds (Figure S5b), the experimental-only model achieved $R^2 = 0.72$, MAE = 0.66 log units, and Spearman $\rho = 0.91$. The increased scatter relative to the internal validation is expected, as the non-experimental compounds include subclasses (zwitterionic, cationic, phosphonic PFAS) not represented in the experimental training set. Despite this, the rank correlation of $\rho = 0.91$ indicates that the experimental-only model preserves the relative ordering of compounds across the full structural diversity of the database.

Most importantly, the experimental-only model preserves the monotonic increase of log KOC with chain length for both PFCAs (C4–C13) and PFSAs (C4–C13) (Figure S5c). The predicted chain-length increments (approximately +0.3 to +0.5 log units per CF_2 for PFCAs and +0.3 to +0.6 for PFSAs) are somewhat compressed relative to the literature values (which show +0.6 and +0.8 per CF_2, respectively), reflecting the limited training range. However, the trend direction is correct, no inversions occur, and the PFSA > PFCA differentiation at the same chain length is maintained.

The EU-20 KOC ranking from the experimental-only model correlates with the full-model ranking at Spearman $\rho = 0.994$, meaning that 19 of 20 compounds occupy the same or immediately adjacent rank position. The single discrepancy involves PFHpA and PFHxS swapping positions 7 and 8 — the same pair identified as borderline in the weight sensitivity analysis (Section S1.3). This near-perfect rank preservation confirms that the regulatory conclusions of the study — which compounds are most and least mobile — are not an artifact of training on predicted data.

S2.4. Conclusion

The experimental-only analysis demonstrates that a model trained exclusively on 17 measured log KOC values captures the fundamental structure–sorption relationships governing PFAS mobility and produces an EU-20 ranking functionally identical to the full model. The inclusion of OPERA and COSMOtherm values in the main model therefore serves to extend the applicability domain to underrepresented subclasses rather than to inflate performance metrics. The circularity concern, while legitimate in principle, does not affect the regulatory conclusions of this work.

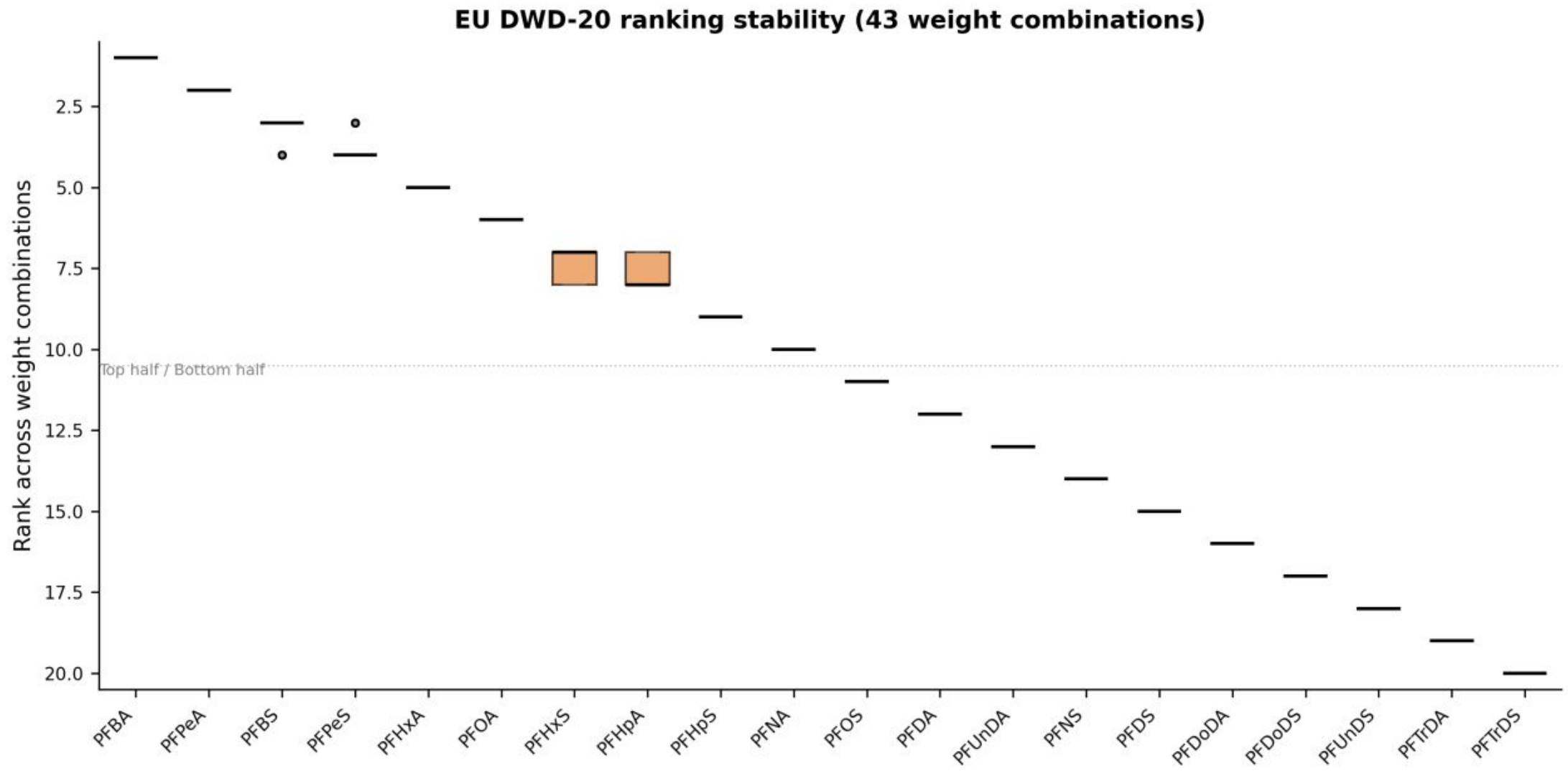

Figure S1. Ranking stability of EU DWD-20 PFAS across 43 GMI weight combinations. Each boxplot shows the distribution of ranks for one compound. Compounds are ordered by mean rank (most mobile at left). Red = high mobility, amber = moderate, green = low. Most compounds show zero rank variation (collapsed boxes), indicating complete insensitivity to weight choice. The only variation occurs for two compound pairs (PFBS/PFPeS and PFHxS/PFHpA) that occasionally swap adjacent positions.

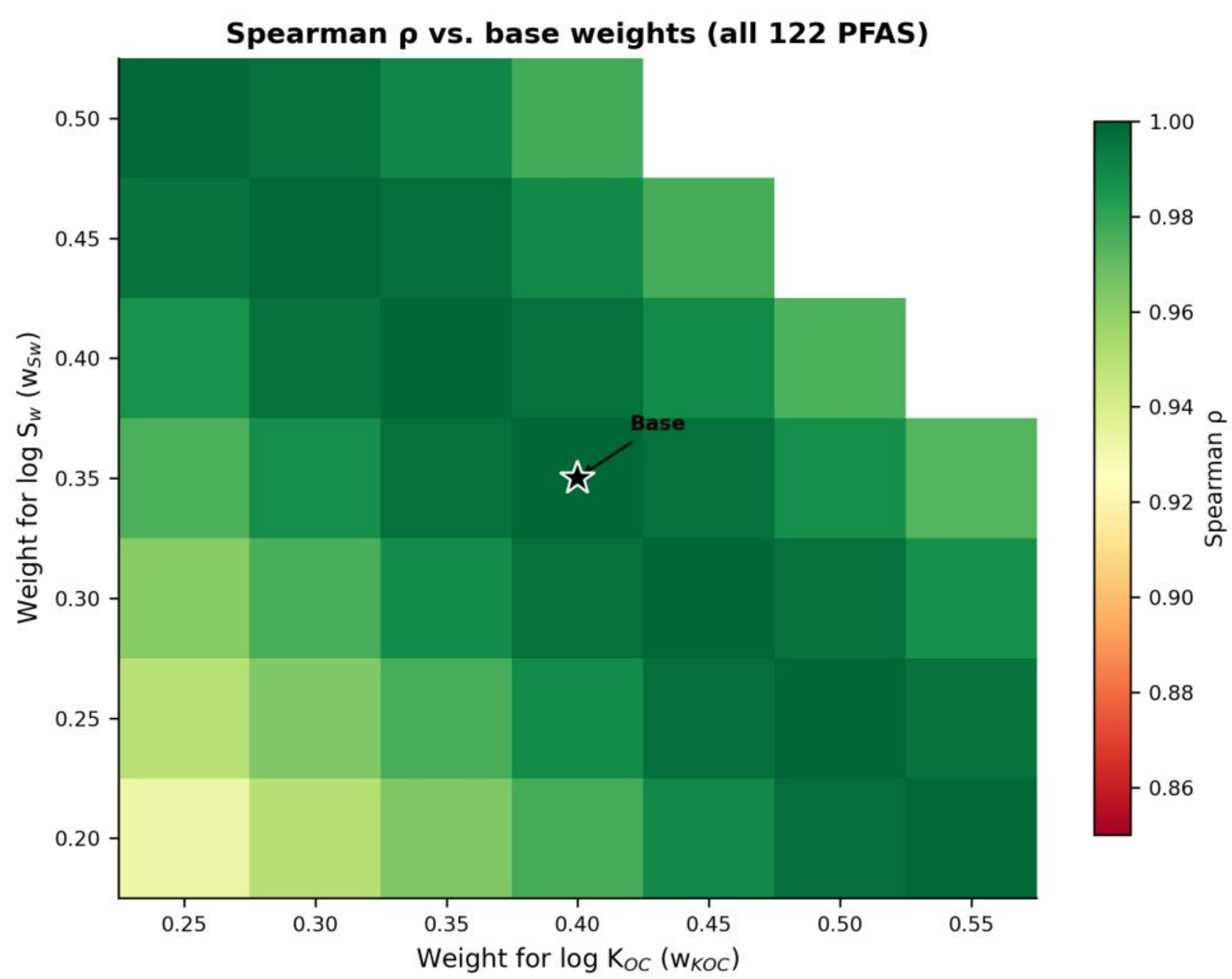

Figure S2. Spearman rank correlation coefficient (ρ) between the base-weight GMI ranking and rankings obtained under each alternative weight combination, mapped as a function of w_{KOC} (x-axis) and w_{Sw} (y-axis). The third weight (w_{ion}) is determined by the constraint $w_{KOC} + w_{Sw} + w_{ion} = 1$. Star marks the base weights (0.40, 0.35, 0.25). All combinations exceed ρ = 0.93; the ranking is most sensitive when ionization weight is very high (upper-left corner, where w_{KOC} and w_{Sw} are both low).

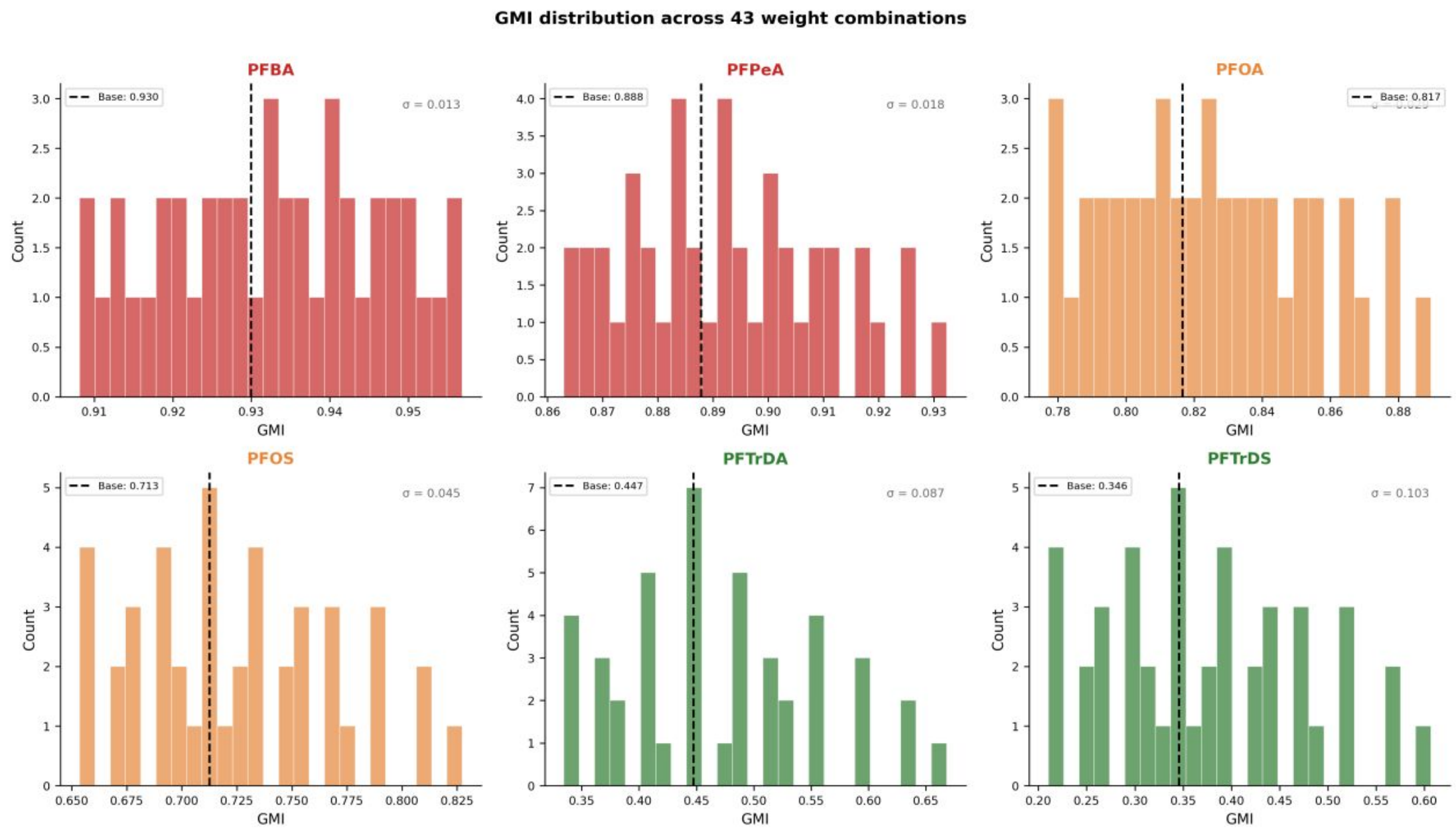

Figure S3. Distribution of GMI values across 43 weight combinations for six representative EU DWD-20 compounds. Top row: highly mobile compounds (PFBA, PFPeA); middle: moderately mobile (PFOA, PFOS); bottom: low mobility (PFTrDA, PFTrDS). Dashed line marks the base-weight GMI. Standard deviations (σ) are reported in each panel. The narrow distributions confirm that GMI values are insensitive to weight perturbation, particularly for compounds at the extremes of the ranking.

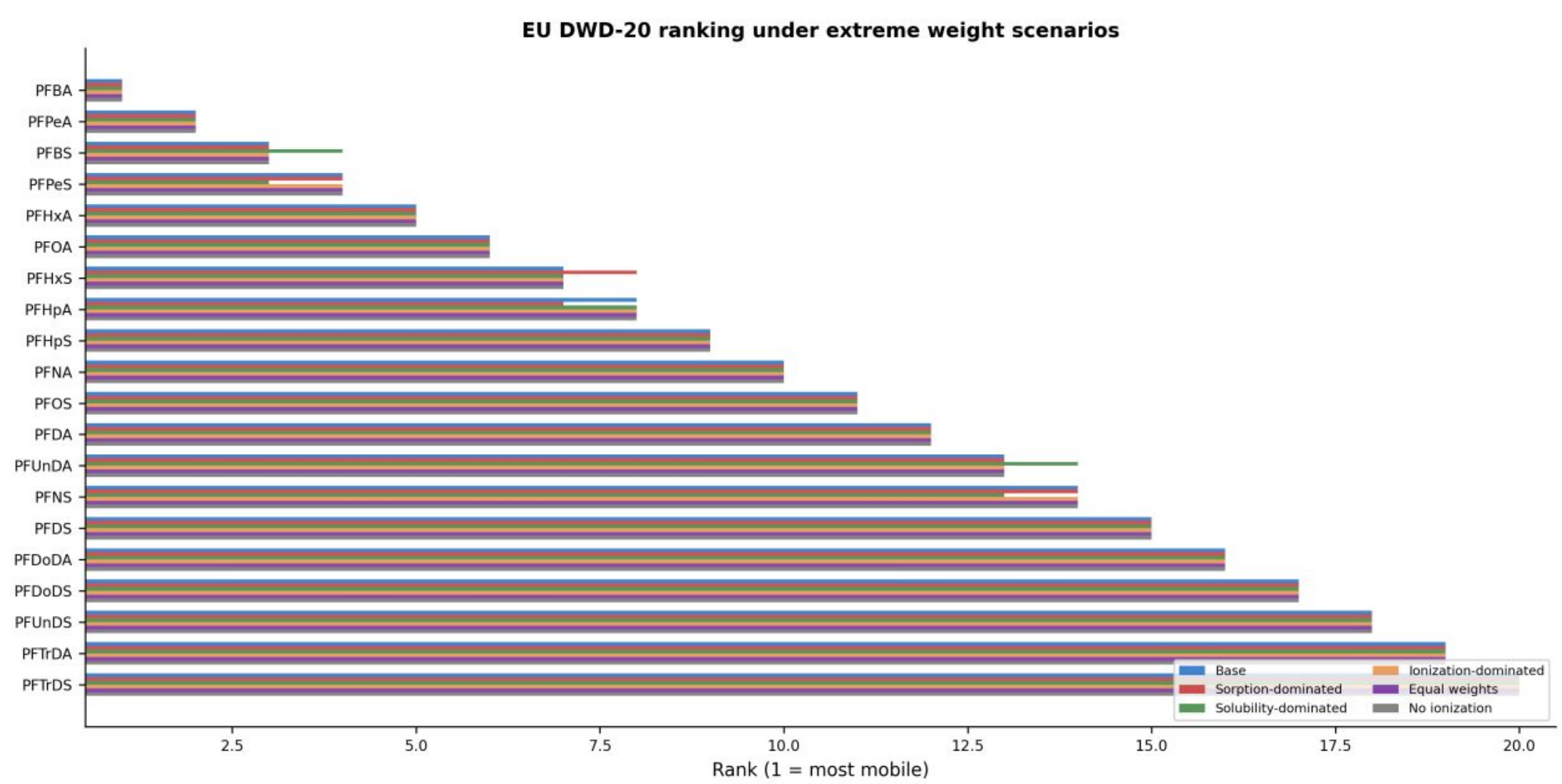

Figure S4. EU DWD-20 compound rankings under six extreme weight scenarios: base (0.40/0.35/0.25), sorption-dominated (0.70/0.15/0.15), solubility-dominated (0.15/0.70/0.15), ionization-dominated (0.15/0.15/0.70), equal weights (0.33/0.34/0.33), and no ionization (0.55/0.45/0.00). Each bar shows the rank assigned under one scenario. Compounds at the top and bottom of the ranking are invariant across all scenarios. Minor reordering occurs only for compounds with nearly identical GMI values.

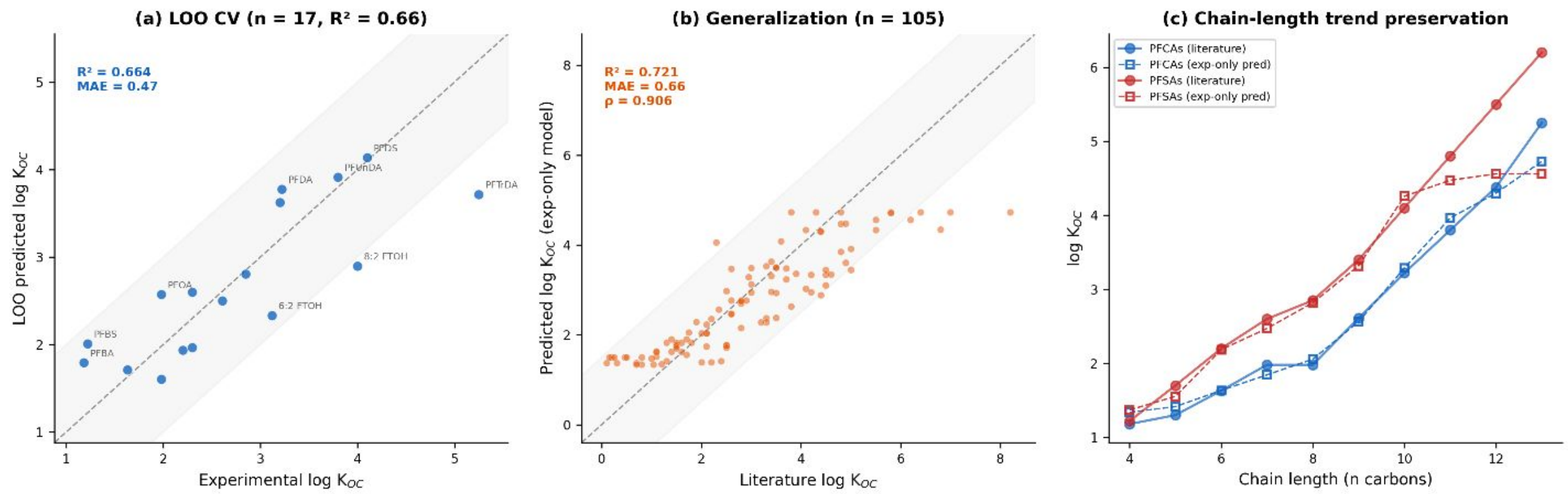

Figure S5. Experimental-only log KOC model trained on 17 compounds with measured sorption coefficients. (a) Leave-one-out cross-validation: each point is one experimental compound predicted from the remaining 16 ($R^2 = 0.66$, MAE = 0.47). Grey band = ±1.0 log unit. (b) External prediction of 105 non-experimental compounds ($R^2 = 0.72$, Spearman $\varrho = 0.91$). (c) Chain-length trend preservation for EU DWD-20 PFCAs (blue) and PFSAs (red): solid lines = literature values, dashed lines with open symbols = experimental-only model predictions. The monotonic increase with chain length is preserved for both series across C4–C13.

S3. Chain-Topology Descriptors and Subclass Generalisation

Leave-one-subclass-out testing of the original 35-descriptor model showed near-zero explained variance for the perfluoroalkyl ether carboxylic acids. Inspection of the descriptor set indicated that no variable represented the structural feature distinguishing these compounds: an ether oxygen interrupts the contiguous hydrophobic perfluoroalkyl surface that drives partitioning to organic matter, so a C8 ether acid behaves not as a C8 perfluoroalkyl acid but as two shorter segments. Nine descriptors were therefore added.

The molecular graph is cut at every ether oxygen — defined as an oxygen bonded to two carbons, bearing no hydrogen, and not adjacent to a carbonyl — and the resulting carbon fragments are scored for the number of perfluorinated carbons they contain (a carbon bearing two or more fluorines). From this decomposition the descriptors in Table S3 are computed.

Table S3. Computed descriptors and their description.

Descriptor	Definition
max_pfa_segment	Number of perfluorinated carbons in the longest contiguous segment
n_pfa_segments	Count of distinct perfluorinated segments after cutting at ether oxygens
mean_pfa_segment	Mean perfluorinated-carbon count per segment
pfa_segment_std	Standard deviation of segment lengths (asymmetry of segmentation)
ether_O_frac	Ether oxygens per carbon atom
O_C_ratio	Total oxygen atoms per carbon atom
n_CF3_term	Number of terminal CF3 groups
ether_head_dist	Topological bond distance from the acid head group to the nearest ether oxygen
branch_at_ether	Number of branch points on carbons adjacent to an ether oxygen

Table 1 of the main text reports the resulting leave-one-subclass-out performance. The subclass-level comparison between the initial and extended descriptor sets is shown in Figure S6. The mean LOSO coefficient of determination for log KOC rose from 0.68 to 0.76 and for log Sw from 0.37 to 0.47, with global five-fold cross-validated performance unchanged (log KOC R^2 0.893 → 0.904; log Sw R^2 0.898 → 0.899), confirming that the gain reflects improved representation rather than additional flexibility being used to fit noise.

Panel (c) of Figure S6 addresses the interpretation of the residual PFECA result. The coefficient of determination is normalised by the variance of the held-out set and is therefore not comparable between subclasses of differing spread. The PFECAs span 1.9 log units of log KOC (SD = 0.54) against 7.7 log units for the PFCAs (SD = 2.42). Judged on mean absolute error, the held-out PFECA predictions (0.35 log units) are the third most accurate of the six subclasses, ahead of the PFCAs (0.50) and PFSAs (0.57) whose R^2 values exceed 0.84.

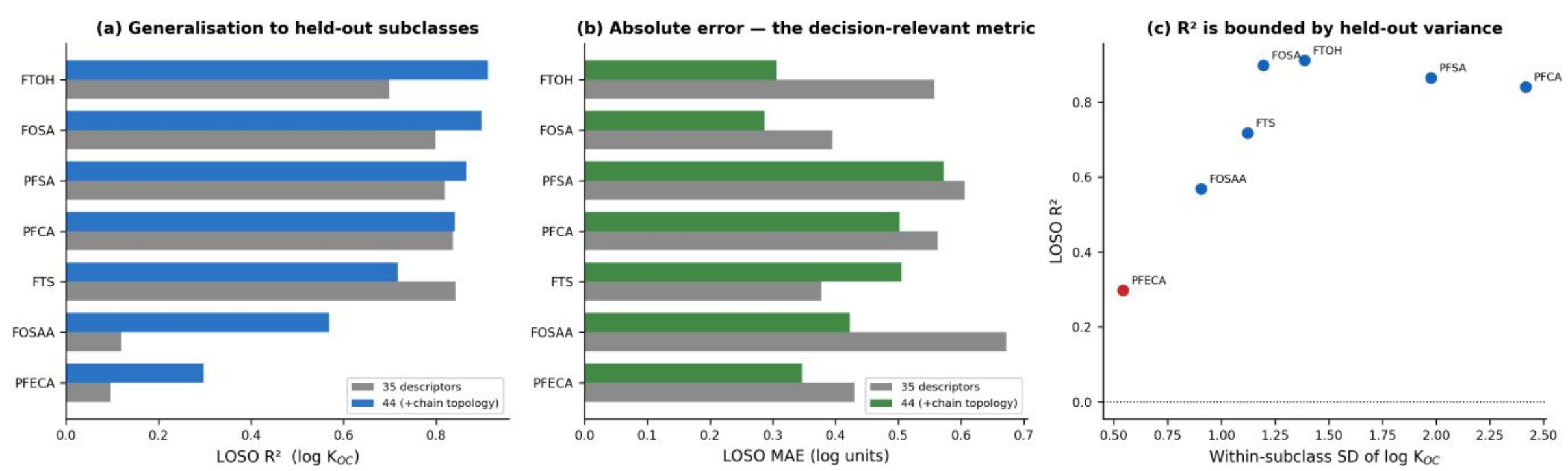

Figure S6. Subclass generalisation before and after adding chain-topology descriptors. (a) Leave-one-subclass-out R^2 for log KOC. (b) Mean absolute error for the same folds. (c) LOSO R^2 plotted against the within-subclass standard deviation of log KOC, showing that the attainable R^2 is bounded by the spread of the held-out set; PFECA in red.

S4. Chance Correlation and Descriptor-Count Sensitivity

Two controls were performed to establish that the reported accuracy does not arise from chance correlation and does not depend on the size of the descriptor set.

S4.1 Y-randomisation. The descriptor matrix was held fixed and the response vector randomly permuted 100 times; a model was retrained and five-fold cross-validated on each permutation. For log KOC the true model achieves $R^2 = 0.904$ against a permuted mean of -0.475 ± 0.157 (maximum -0.069), giving Z = 8.8. For log Sw the corresponding values are $R^2 = 0.899$ against -0.473 ± 0.149 (maximum -0.149), Z = 9.2. No permuted model approached the true performance in either case (empirical $p < 0.01$).

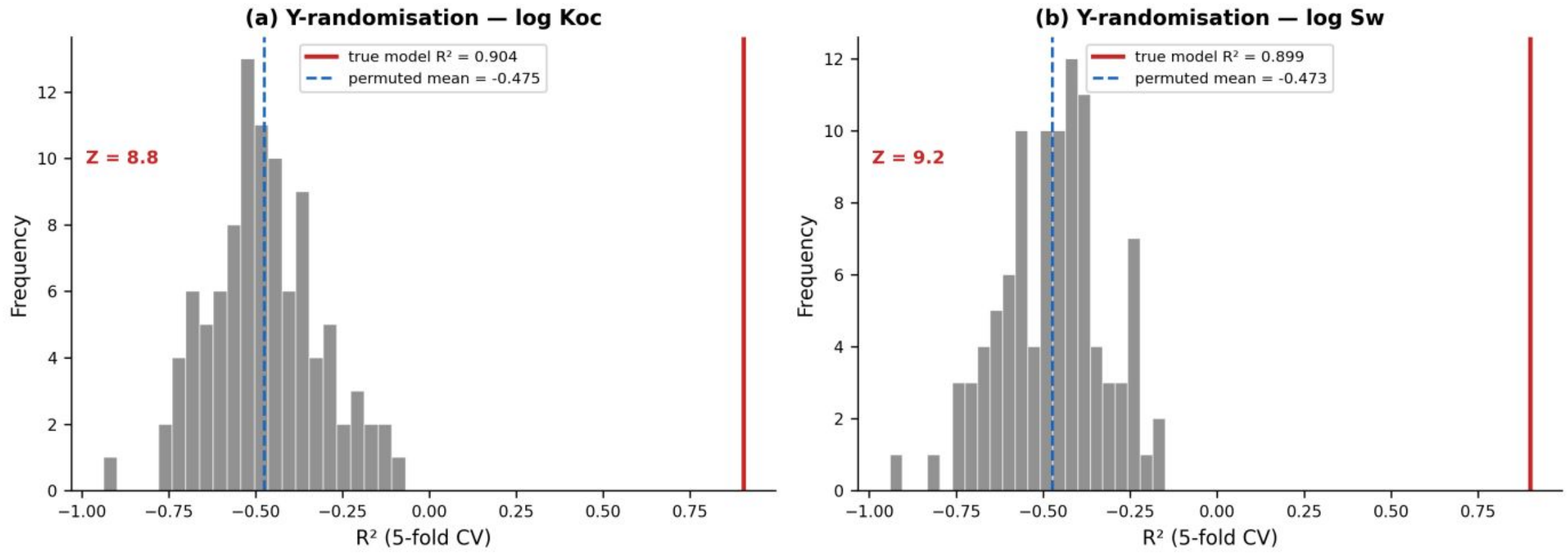

Figure S7. Y-randomisation. Distribution of cross-validated R^2 over 100 label permutations (grey) against the true model (red). (a) log KOC. (b) log Sw.

S4.2 Descriptor-count ablation. Descriptors were ranked by gain-based importance and models retrained on the top k for k = 5 to 44. Performance rises steeply to approximately 15 descriptors and then plateaus: for log KOC, $R^2 = 0.897$ at $k = 15$ against 0.911 at $k = 35$ and 0.907 at $k = 44$. The model therefore does not depend on the larger descriptor count, and the compound-to-descriptor ratio at the plateau is approximately 8:1.

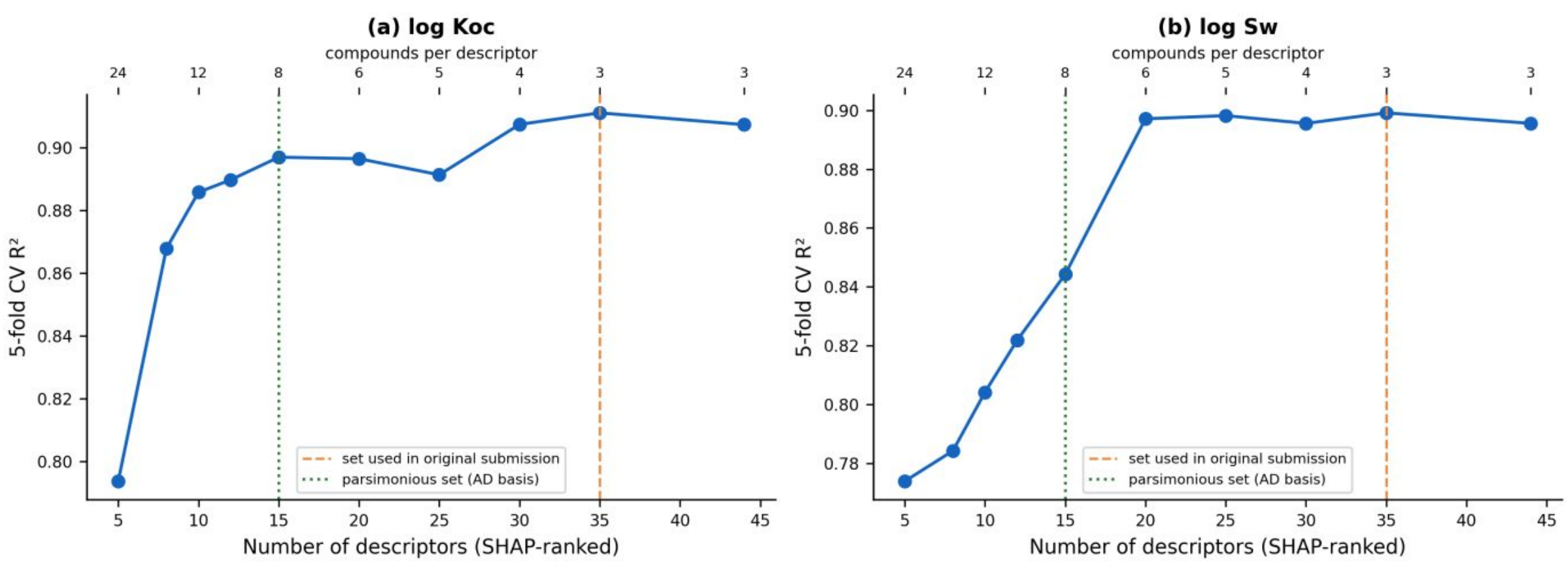

Figure S8. Five-fold cross-validated R^2 as a function of the number of descriptors retained. Upper axis gives the corresponding compounds-per-descriptor ratio.

S5. Applicability Domain

A formal applicability domain was defined because the framework is distributed for use on compounds outside the training set. Two criteria are applied jointly.

Leverage. $h_i = x_i^T(X^TX)^{-1}x_i$ is evaluated on the 15 highest-ranked descriptors, with the conventional threshold $h^* = 3(p+1)/n = 0.393$. A reduced basis is necessary: with all 44 descriptors $h^* = 1.107$, exceeding unity, and the criterion becomes undefined. Six compounds exceed the leverage threshold and two exceed three standardised residuals, giving 8 of 122 outside the domain.

Nearest-neighbour distance. The mean Euclidean distance to the five nearest training neighbours in standardised descriptor space is compared with the 95th percentile of the training distribution (3.21).

Descriptor-space coverage differs markedly between subclasses (Figure S9b). The perfluoroalkyl acids, fluorotelomer alcohols and sulfonamides are densely sampled; the perfluoroalkyl phosphinic acids, partially fluorinated compounds and zwitterionic and cationic species occupy sparse regions with nearest-neighbour distances two to three times the median. The prediction tool applies both criteria to every query and returns an out-of-domain warning, withholding the numerical GMI, when either is violated.

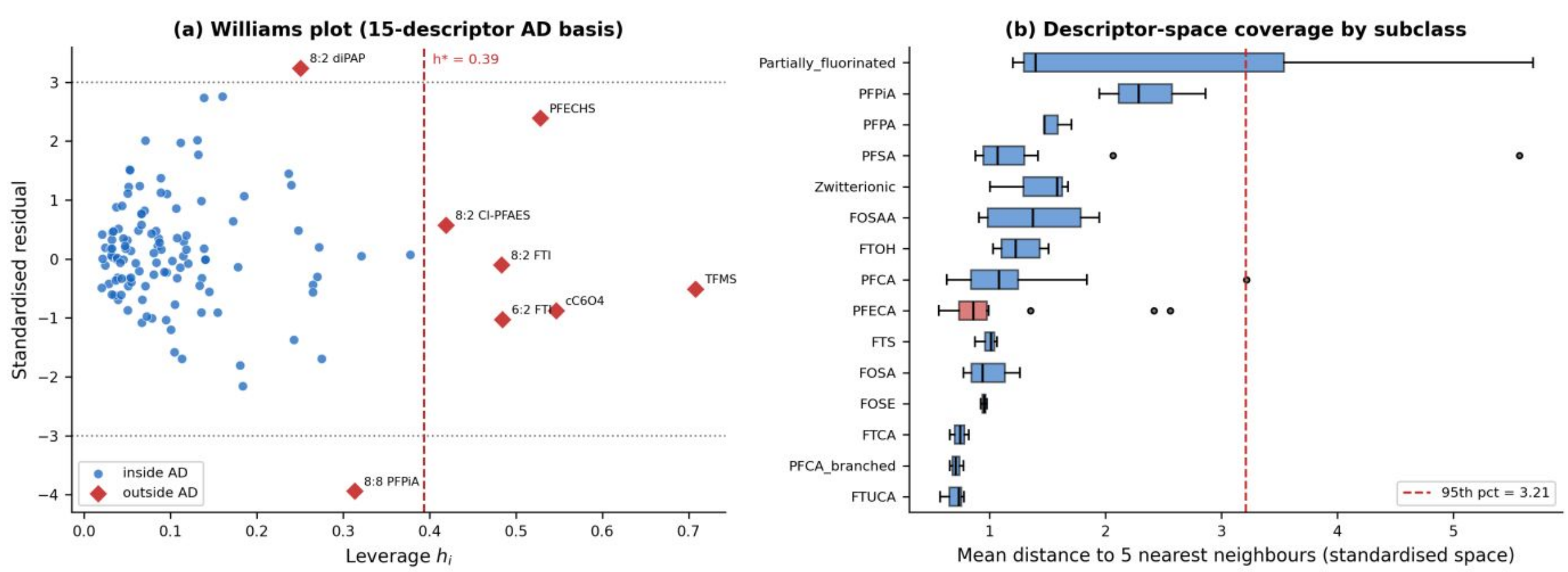

Figure S9. Applicability domain. (a) Williams plot on the 15-descriptor basis; compounds outside the domain in red. (b) Distribution of mean distance to the five nearest neighbours by subclass; PFECA in red.

S6. GMI Normalisation and Weight Derivation

S6.1 Absolute normalisation. The dataset-relative min–max scheme used in the initial analysis makes a GMI value meaningful only within the compound set from which it was computed. Appending a single hypothetical ultra-mobile compound to the database shifts the normalised sorption score of existing compounds by up to 0.177 units. An absolute scheme was therefore adopted, anchoring log KOC to [0, 7] log L kg^{-1} and log Sw to [−4, 6] log mg L^{-1}, bounds that span the reported envelope of PFAS behaviour, with clipping outside. Scores on this scale are

independent of database composition. The two schemes give almost identical orderings (Spearman ϱ = 0.9994 over all 122 compounds; ϱ = 1.0000 within the EU-20, with an identical top-five ranking).

S6.2 Weight derivation. The adopted weights of 0.40, 0.35 and 0.25 are an expert-judgement allocation. Two independent derivations were performed as corroboration.

Table S4. Independent derivations of the GMI weights and the rank correlation of the resulting rankings with the adopted allocation.

Derivation	w(log KOC)	w(log Sw)	w(ionisation)	ϱ vs adopted (all)	ϱ vs adopted (EU-20)
Expert judgement (adopted)	0.40	0.35	0.25	—	—
Transport sensitivity	0.33	0.41	0.27	0.9992	1.0000
Shannon entropy	0.19	0.15	0.66	0.9144	1.0000

The transport-sensitivity derivation evaluates the elasticity of solute velocity with respect to each property using the retardation factor $R = 1 + \varrho_b f_{oc} KOC / \theta$ over f_{oc} = 0.001–0.05 and normalises the result. It arrives at an allocation close to the adopted one from transport physics rather than judgement. The Shannon-entropy derivation, a standard objective multi-criteria procedure, produces a markedly different allocation that nevertheless reproduces the EU-20 ranking exactly.

S6.3 Soil context. The rank correlation between the retardation-factor ordering and the GMI ordering remains $|\varrho|$ = -0.994 across f_{oc} = 0.001–0.05, a two-order-of-magnitude range, while R itself varies by two orders of magnitude. Organic carbon content therefore rescales absolute travel time without reordering compounds. Soil pH is not captured by the framework and is not benign in this respect, as discussed in Section 3.6 of the main text.

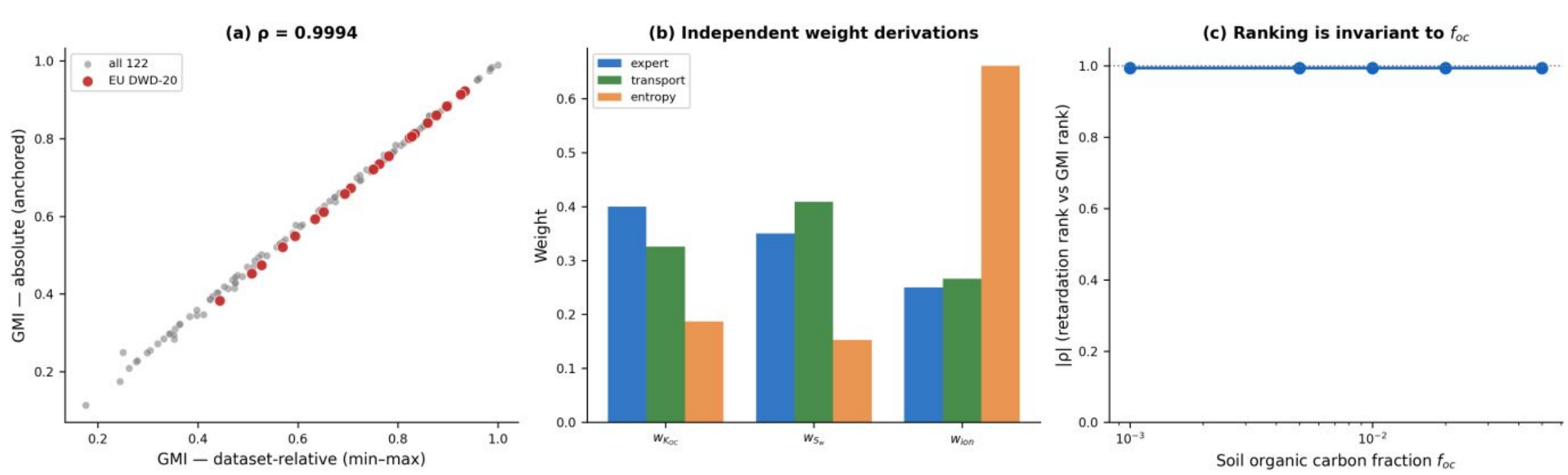

Figure S10. (a) Absolute against dataset-relative GMI. (b) Weight allocations from three derivations. (c) Rank correlation between retardation-factor and GMI orderings as a function of soil organic carbon fraction.

S7. Extended Experimental-Only Analysis

Section S2 of trained a log KOC model on the 17 compounds with measured sorption coefficients and evaluated it on the EU-20. Two limitations of that analysis were identified: it covered only one endpoint, and the EU-20 are twenty homologues following a monotonic chain-length trend, so rank stability among them is a weak test. The analysis was therefore repeated for all three endpoints and evaluated on the compounds outside the perfluoroalkyl acid families.

Table S5. Experimental-only models for all three endpoints. The pKa row is reported for completeness but is not interpretable: see below.

Endpoint	n measured	LOO R^2	All non-experimental	Non-PFAA subclasses
log KOC	17	0.758	$R^2 = 0.718$, $\varrho = 0.910$	$R^2 = 0.693$, $\varrho = 0.878$ (n = 85)
log Sw	15	0.554	$R^2 = 0.499$, $\varrho = 0.840$	$R^2 = 0.505$, $\varrho = 0.820$ (n = 83)
pKa class	17	acc = 0.765	acc = 0.524	acc = 0.390 (n = 82)

The log KOC result supports the main model. A model that has seen only 17 measured values predicts the 85 compounds outside the perfluoroalkyl acid families with $\varrho = 0.878$, so the sorption ranking is not an artefact of circular training and this holds beyond the homologous series. The log Sw result is weaker; absolute solubility predictions carry greater uncertainty than sorption predictions, although the rank information used by the GMI is preserved.

The pKa endpoint cannot be tested this way. The 17 compounds with measured or NMR-determined pKa comprise 13 strong acids and 4 weak acids and contain no neutral-class member, because the experimental literature has concentrated on the ionisable perfluoroalkyl acids. A model trained on that subset cannot predict a class it has never encountered, and the apparent accuracies in the final row of Table S7b reflect that absence rather than model quality.

An alternative check was therefore performed. Class labels were assigned purely from functional-group identity using SMARTS patterns, with no fitted parameters and no reference to any model or thermodynamic calculation: presence of a sulfonic or phosphonic acid, or a carboxylic acid on a perfluorinated α-carbon, gives the strong-acid class; a carboxylic acid on a non-perfluorinated α-carbon gives the weak-acid class; an alcohol or sulfonamide with no ionisable acid gives the neutral class. This rule-based assignment reproduces the database labels for 80.3% of all compounds, and specifically for 93.4% of strong acids and 89.7% of neutrals, but for only 46.9% of the intermediate weak-acid class. The two classes carrying the extreme ionisation scores are therefore largely determined by structure rather than by any model, whereas the intermediate class is genuinely uncertain. All twenty EU DWD substances fall in the strong-acid class.

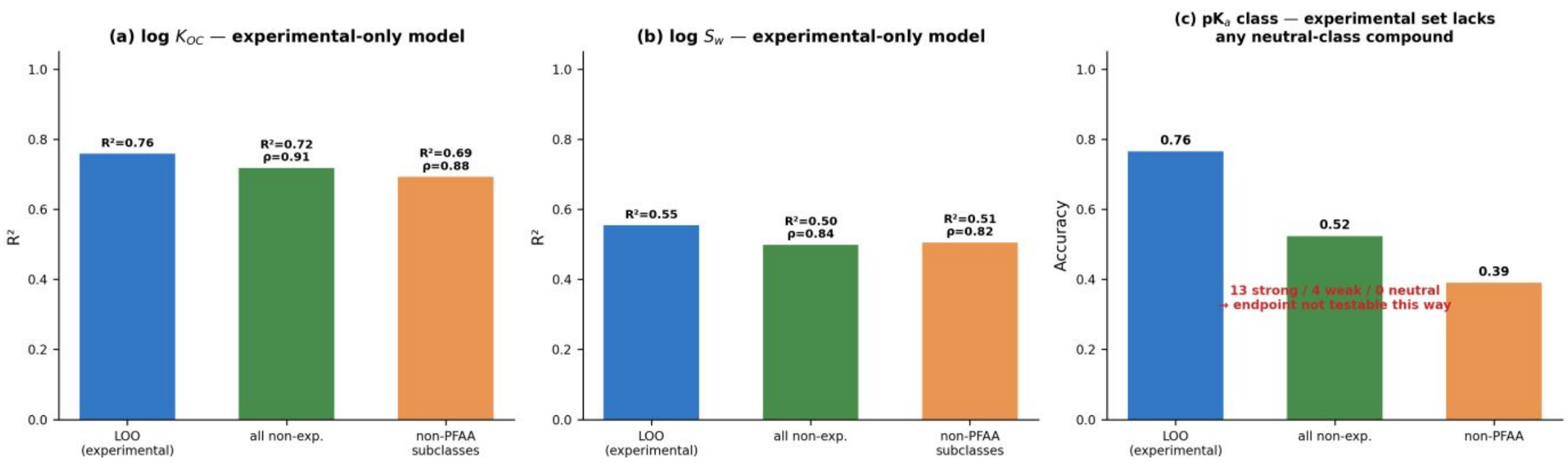

Figure S11. Experimental-only models. (a) log KOC. (b) log Sw. (c) pKa class, where the experimental subset contains no neutral-class compound and the test is therefore not interpretable.

S8. Benchmark Comparison with Existing Tools

Table S6. Comparison of the present framework with existing predictive tools for PFAS environmental fate.

	**This work**	**PFASorptionML**	**OPERA / EPI Suite**	**COSMOtherm**
Input required	SMILES only	SMILES + measured soil properties	SMILES	Optimised 3D conformer ensemble
Endpoints returned	log KOC, log Sw, pKa class	Kd	Sw, logP (single)	Partition coefficients
PFAS-specific training	Yes (122 PFAS, 37 subclasses)	Yes (1,274 entries)	No (mainly non-fluorinated)	Not applicable (first principles)
Joint mobility profile	Yes	No	No	No
Ranked regulatory output	Yes (GMI)	No	No	No
Applicability domain enforced	Yes	Partial	Yes	Not applicable
Expertise required of user	None	Soil characterisation	None	Computational chemistry
Cost per compound	Seconds, free	Seconds, free	Seconds, free	Hours, licensed
