# Supplementary material for: Structure-Based Prediction and Ranking of PFAS Groundwater Mobility for EU Drinking Water Regulation
Source: Environ Sci Technol. 2026 Sep 29;60(40):29077–88. doi: 10.1021/acs.est.6c11513 (PMC13674058; doi:10.1021/acs.est.6c11513)
Supplement: Supplementary file 2 [file es6c11513_si_004.zip › pfas_predictor_tool_v2.html]

PFAS Groundwater Mobility Predictor v2.0

# PFAS Groundwater Mobility Predictor

Aparicio et al., Environmental Science & Technology 2026

v2.0 · absolute normalisation · applicability-domain screening

### Scope — please read before use

**Appropriate for:**

- Screening-level prioritisation of perfluoroalkyl acids and structurally similar compounds inside the applicability domain
- Comparative ranking of candidate replacement chemistries
- Identifying compounds that warrant experimental follow-up

**Not appropriate for:**

- Site-specific exposure or risk assessment
- Compounds flagged out-of-domain — no GMI is issued for these
- Polymeric or precursor PFAS governed by transformation rather than partitioning
- Regulatory determination on an individual substance without supporting measurement

The index describes **intrinsic** mobility, a property of the molecule. It does not account for
soil pH, ionic strength, mineralogy or air–water interfacial adsorption. Organic carbon content
rescales travel time but does not reorder compounds (|ρ| = 0.994 across f_oc_ = 0.001–0.05);
soil pH *can* reorder ionisable compounds.

**122**compounds

**—**inside applicability domain

**20**EU DWD Annex III

**44**descriptors

All compoundsEU DWD-20 only
Inside domain onlyOut of domain only
All mobility bandsVery high
HighModerateLow
Very low

| Compound | Subclass | log K_OC_ | log S_w_ | Ionization | GMI | Mobility | Domain |
| --- | --- | --- | --- | --- | --- | --- | --- |

Click a column header to sort. GMI uses absolute normalisation anchored to
log K_OC_ ∈ [0, 7] and log S_w_ ∈ [−4, 6], so scores are independent
of database composition. To predict a **new** compound from its SMILES, use the Python
application (`pfas_predictor_app_v2.py`), which computes descriptors with RDKit and applies
the same applicability-domain test.
